# Identification and Functional Characterization of Peptides With Antimicrobial Activity From the Syphilis Spirochete, *Treponema pallidum*

**DOI:** 10.3389/fmicb.2022.888525

**Published:** 2022-05-03

**Authors:** Simon Houston, Ethan Schovanek, Kate M. E. Conway, Sarah Mustafa, Alloysius Gomez, Raghavendran Ramaswamy, Ayman Haimour, Martin J. Boulanger, Lisa A. Reynolds, Caroline E. Cameron

**Affiliations:** ^1^Department of Biochemistry and Microbiology, University of Victoria, Victoria, BC, Canada; ^2^Division of Allergy and Infectious Diseases, Department of Medicine, University of Washington, Seattle, WA, United States

**Keywords:** antimicrobial peptides, syphilis, *Treponema pallidum*, bacteriostatic, bactericidal

## Abstract

The etiological agent of syphilis, *Treponema pallidum* ssp. *pallidum*, is a highly invasive “stealth” pathogen that can evade the host immune response and persist within the host for decades. This obligate human pathogen is adept at establishing infection and surviving at sites within the host that have a multitude of competing microbes, sometimes including pathogens. One survival strategy employed by bacteria found at polymicrobial sites is elimination of competing microorganisms by production of antimicrobial peptides (AMPs). Antimicrobial peptides are low molecular weight proteins (miniproteins) that function directly via inhibition and killing of microbes and/or indirectly via modulation of the host immune response, which can facilitate immune evasion. In the current study, we used bioinformatics to show that approximately 7% of the *T. pallidum* proteome is comprised of miniproteins of 150 amino acids or less with unknown functions. To investigate the possibility that AMP production is an unrecognized defense strategy used by *T. pallidum* during infection, we developed a bioinformatics pipeline to analyze the complement of *T. pallidum* miniproteins of unknown function for the identification of potential AMPs. This analysis identified 45 *T. pallidum* AMP candidates; of these, Tp0451a and Tp0749 were subjected to further bioinformatic analyses to identify AMP critical core regions (AMPCCRs). Four potential AMPCCRs from the two predicted AMPs were identified and peptides corresponding to these AMPCCRs were experimentally confirmed to exhibit bacteriostatic and bactericidal activity against a panel of biologically relevant Gram-positive and Gram-negative bacteria. Immunomodulation assays performed under inflammatory conditions demonstrated that one of the AMPCCRs was also capable of differentially regulating expression of two pro-inflammatory chemokines [monocyte chemoattractant protein-1 (MCP-1) and interleukin-8 (IL-8)]. These findings demonstrate proof-of-concept for our developed AMP identification pipeline and are consistent with the novel concept that *T. pallidum* expresses AMPs to defend against competing microbes and modulate the host immune response.

## Introduction

The spirochete bacterium, *Treponema pallidum* ssp. *pallidum* (hereafter *T. pallidum*), is the causative agent of syphilis, a chronic, multistage infection that is transmitted sexually or in utero. Following infection, *T. pallidum* traverses endothelial barriers and undergoes rapid and widespread dissemination via the circulatory system to infect every organ and tissue, including immunologically privileged sites such as the eyes (Marra et al., 1991; Muller et al., 2007), testes (Sell et al., 1980), and central nervous system (Collart et al., 1971; Lukehart et al., 1988). Despite host-initiated innate and adaptive immune responses, *T. pallidum* is able to persist within the host for decades (Lafond and Lukehart, 2006). The remarkable ability of *T. pallidum* to evade the immune system and establish and maintain persistent infection has earned it the designation of the “stealth” pathogen (Radolf, 1994).

During infection of a host, *T. pallidum*, which has a slow generation time of 30–33 h (Magnuson and Eagle, 1948; Cumberland and Turner, 1949), are introduced into anatomical sites that are abundant in species of microbiota, including the genital tract, skin, rectum, and oral cavity (Lafond and Lukehart, 2006), features that may put *T. pallidum* at a growth disadvantage in a polymicrobial environment. In addition, *T. pallidum* can present as a co-infection with other viral, fungal, parasitic, and bacterial pathogens, including the sexually transmitted pathogen, *Neisseria gonorrhoeae* (Bala et al., 2011; World Health Organization, 2019; Pinho-Bandeira et al., 2020; Coelho et al., 2021). Inhibition and elimination of competing microorganisms via production of antimicrobial peptides (AMPs) allows the microbiota and pathogenic bacteria found at polymicrobial sites to gain a competitive advantage (Meade et al., 2020). The ability of *T. pallidum* to establish an infection and survive in anatomical locations with a complex polymicrobial profile raised the question of whether this bacterium could use AMP production to eliminate microbial competition.

AMPs are a structurally and functionally diverse class of low molecular weight proteins produced by all branches of life (Kumar et al., 2018). Typically comprised of 10–150 amino acids, they often form amphipathic alpha helices facilitated by their net positive charge and high hydrophobic content (Kumar et al., 2018). AMPs have a direct mechanism of action that occurs via electrostatic interactions between positively-charged AMPs and negatively-charged microbial surfaces (Kumar et al., 2018). The amphipathic secondary structure of AMPs promotes membrane integration and pore formation, resulting in membrane destabilization and cell lysis as well as inhibition of essential intracellular functions such as DNA and protein synthesis (Kumar et al., 2018; Mishra et al., 2018). A second, indirect effect of AMPs can be alteration of the host immune response, including modulation of inflammatory cytokine production, immune cell recruitment and activation (Hilchie et al., 2013). The immunomodulatory effects of eukaryotic AMPs have been well documented, and recent studies have shown bacterial AMPs can have similar immunomodulatory activities (Kindrachuk et al., 2013; Malaczewska et al., 2019) that can promote bacterial survival and host infection via subversion and evasion of the host immune response (Li et al., 2014).

An important characteristic of AMPs is the presence of functionally essential regions that correspond to the shortest stretch of amino acids (often ∼10–20 residues) that retain antimicrobial effects (Chang et al., 2015). Identification of these key regions, defined as antimicrobial peptide critical core regions (AMPCCRs), allows for the design and development of discrete peptides with antimicrobial activity that are derived from, and more tractable than, their larger precursor proteins (Torrent et al., 2009, 2012).

More than two decades have passed since the first *T. pallidum* whole genome sequence was published (Fraser et al., 1998). Since then, many laboratory and clinical strains of *T. pallidum* ssp. *pallidum* have been sequenced, yet only three genes have been annotated as homologs of known AMP-related genes in other bacteria. These are *tp0688* [*Bacillus anthracis mccF*, encoding the Microcin C7 self-immunity protein (Gonzalez-Pastor et al., 1995)], *tp0522* [*Escherichia coli cvpA*, encoding the Colicin V Production protein that is required for production and secretion of the AMP Colicin V (Gilson et al., 1990)], and *tp0405* [*E. coli mcbG*, encoding the Microcin B17 self-immunity protein (San Millan et al., 1985)]. Recent *T. pallidum* genome sequencing also identified a novel 91-amino acid miniprotein (TPANIC_RS05485) which has been annotated as a putative CPBP (CAAX Protease and Bacteriocin-Processing) family intramembrane metalloprotease. Evidence suggests some members of this family may be involved in bacterial AMP processing (Pei et al., 2011). One reason that may partially account for the low number of AMP-related genes detected within *T. pallidum* to date is that the bacterium is phylogenetically distinct, with approximately 300 genes/30% of the genome predicted to encode proteins with no known orthologs or assigned functions (Fraser et al., 1998; Petrosova et al., 2013).

The present study shows that approximately one quarter of *T. pallidum* genes of unknown function are predicted to encode miniproteins of 150 amino acids or less. Bioinformatic analyses show a portion of these miniproteins possess characteristics consistent with known AMPs. These findings, when considered in the context of the success of this bacterium at establishing infection at polymicrobial anatomical sites, prompted us to investigate whether AMP production is an unexplored pathogenic mechanism used by *T. pallidum* to defend against competing microbes and the host. Herein, we investigated this potential treponemal defense strategy using a combination of bioinformatics, structure modeling, antimicrobial susceptibility testing, and immunomodulation assays. Our investigations have provided experimental confirmation of AMP activity within two *T. pallidum* miniproteins, consistent with the novel concept that *T. pallidum* expresses AMPs to establish and maintain infection at polymicrobial sites in the human host.

## Materials and Methods

### Bacterial Strains and Culture

Bacterial strains used in this study were: *E. coli* ATCC 9723H, *Pseudomonas aeruginosa* ATCC 10148, *Staphylococcus aureus* ATCC 6538P (penicillin resistant), *Streptococcus pyogenes* (hospital isolate, strain unknown), *Mycobacterium smegmatis* MC^2^155, *N. gonorrhoeae* ATCC 700825 (streptomycin resistant), and *Salmonella enterica* subsp. *enterica* serovar Typhimurium SL1344 (streptomycin resistant). *E. coli*, *P. aeruginosa*, *S. aureus*, *M. smegmatis*, and *S. enterica* were cultured aerobically at 37°C in Mueller Hinton broth (MHB) (Sigma-Aldrich, MO, United States) and on nonselective Mueller Hinton agar (MHA) plates. *S. pyogenes* was cultured in 5% carbon dioxide at 37°C in MHB supplemented with 5% lysed horse blood (Quad Five, MT, United States) (MHB + 5% HB) and on nonselective MHA plates supplemented with 5% defibrinated sheep blood (Quad Five, MT, United States) (MHA + 5% SB). *N. gonorrhoeae* was cultured in 5% carbon dioxide at 37°C in gonococcal (GC) chocolate broth medium and on nonselective GC agar plates [GC medium base (BD Difco, MD, United States) supplemented with 1% BBL™ hemoglobin (BD Biosciences, MD, United States) and 1% IsoVitaleX (BD Biosciences, MD, United States)]. Prior to antimicrobial susceptibility assays (as described below), *N. gonorrhoeae* cultures were subcultured on nonselective GC agar plates (as described above), to ensure bacterial viability. All bacterial stocks were stored in 20% glycerol at –80°C.

### *Treponema pallidum* Propagation and *in vitro* Culture

*Treponema pallidum* subsp. *pallidum* (Nichols strain) was propagated in, and extracted from, New Zealand White rabbits as described elsewhere (Lukehart and Marra, 2007), and stored in liquid nitrogen. Frozen treponemal stocks were then used for *in vitro* culture and sub-culture of *T. pallidum* in the presence of Sf1Ep (NBL-11) cottontail rabbit epithelial cells (ATCC CCL-68) [American Type Culture Collection (ATCC), Rockville, MD, United States]. Continuous axenic culture of *T. pallidum* in the absence of mammalian cells has not been achieved, and it is believed that the direct adherence of *T. pallidum* to Sf1Ep cells is required for the long term replication of *T. pallidum in vitro* (Edmondson et al., 2018). Dissociation of *T. pallidum* from Sf1Ep cells was accomplished using trypsin-free dissociation buffer [2 mL: 64% cell culture grade water (Sigma Aldrich), 10% modified EBSS (Earle’s Balanced salt solution, 10×), 1% non-essential amino acids (Thermo Fisher Scientific), 0.15% sodium bicarbonate (Sigma Aldrich), 0.728% 100 mM sodium pyruvate (Sigma Aldrich), 0.136% 0.5M EDTA (Thermo Fisher Scientific), 0.16 mg dithiothreitol (DTT) (Sigma Aldrich)] followed by a low speed centrifugation step (220 × *g*) to separate *T. pallidum* from the rabbit cells, as previously described (Edmondson et al., 2018; Edmondson and Norris, 2021).

### Bioinformatics Pipeline: *In silico* Analysis of *T. pallidum* Whole Proteomes

**FIGURE 1:**
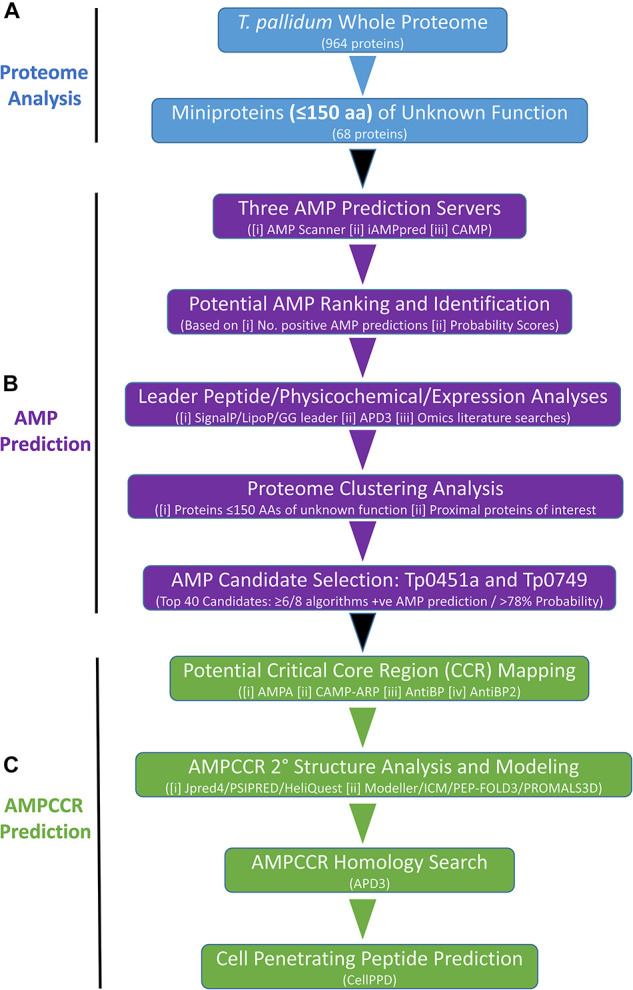
Bioinformatics pipeline for the identification of potential AMPs and AMPCCRs in the proteome of *T. pallidum*. **(A)** Proteome Analysis: The whole proteome (from NCBI genome database, July 2021 annotation) of *T. pallidum* was searched for proteins with no assigned function and 150 amino acids or less. **(B)** AMP Prediction: Complete amino acid sequences of 68 *T. pallidum* proteins (≤150 amino acids) of unknown function were submitted to three AMP prediction servers which allowed for ranking from most likely AMP to least likely AMP. Additional leader peptide, physicochemical, expression, and proteome clustering analyses facilitated the identification of two potential AMPs (Tp0451a and Tp0749) for further analyses. **(C)** AMPCCR prediction: A combination of critical core region (CCR) mapping, structural, homology, and cell penetrating peptide analyses were then performed to further confirm the initial AMP predictions generated by the three prediction servers and to identify putative antimicrobial peptide critical core regions (AMPCCRs) within the identified predicted AMPs, Tp0451a and Tp0749.

The flow diagram shown in Figure 1 outlines all major steps that comprised our bioinformatics pipeline for the identification of potential AMPs and AMPCCRs in the *T. pallidum* proteome. As the first step in this approach, the whole proteome of *T. pallidum* (Nichols strain NC_021490) was obtained from the National Center for Biotechnology Information (NCBI) Genome database^1^ and manually searched in order to identify all functionally-unannotated miniproteins containing 150 amino acids or less (Figure 1A). All *in silico* proteomic analyses performed on the *T. pallidum* strain reported in the current study were based on the NCBI whole proteome annotation from July 2021.

### Bioinformatics Pipeline: AMP Prediction

The full-length amino acid sequences of all functionally-unannotated miniproteins identified in the above analyses were submitted to three AMP prediction servers: (i) AMP Scanner Version 2 (Deep Neural Learning Method for predicting antibacterial activity only)^2^ (Veltri et al., 2018), (ii) iAMPpred [Support Vector Machine (SVM) algorithms for predicting antibacterial, antiviral, and antifungal activities; machine learning method based on amino acid composition, physicochemical, and structural features]^3^ (Meher et al., 2017), and (iii) CAMP [SVM, Random Forest (RF), Artificial Neural Network (ANN), and Discriminant Analysis (DA) algorithms for predicting antimicrobial activity; machine learning method based on different physicochemical properties of proteins]^4^ (Waghu et al., 2016; Figure 1B). All *T. pallidum* miniproteins of unknown function were ranked from most likely AMP to least likely AMP based on the number of different server algorithms (out of eight total) that produced positive AMP predictions. Corresponding mean probability scores (1 [most likely prediction score]-0 [least likely prediction score]) were then used to rank all miniproteins within each positive AMP prediction class (Figure 1B).

### Bioinformatics Pipeline: Leader Peptide, Physicochemical, and Proteome Clustering Analyses of Putative *T. pallidum* AMPs

To determine if any of the miniproteins of unknown function from *T. pallidum* contain potential Sec-dependent leader peptides (Sec/SP1 peptides), we used the signal peptide prediction servers, SignalP 5.0^5^ (Almagro Armenteros et al., 2019) and LipoP 1.0^6^ (Juncker et al., 2003). Manual searches of the *T. pallidum* miniproteins for the conserved double-glycine/glycine-alanine leader peptide motif (M[R/K]ELX_3_E[I/L]X_2_[I/V]XG[G/A]) that has been observed in AMPs from Gram-negative bacteria (Michiels et al., 2001; Dirix et al., 2004) were performed to identify proteins that contain Glycine-Glycine and/or Glycine–Alanine pairs within the first 31 residues of the N-terminus (Figure 1B). A multiple sequence alignment of the N-termini of each of the Glycine-Glycine and/or Glycine-Alanine-containing proteins was generated using Clustal Omega^7^ (Madeira et al., 2019). WebLogo^8^ (Crooks et al., 2004) was then used to generate a sequence logo for the identification of sites within the N-terminal residues of these proteins that exhibit homology to the conserved double-glycine/glycine-alanine leader peptide motif found in AMPs from Gram-negative bacteria (Michiels et al., 2001; Dirix et al., 2004). Physicochemical properties, including hydrophobicity, net charge, and presence/absence of cysteine residues, of the *T. pallidum* miniproteins of unknown function were analyzed using the APD3 database Antimicrobial Peptide Calculator and Predictor^9^ (Wang et al., 2016; Figure 1B). Tertiary structure modeling of functionally-unannotated proteins of interest (>150 amino acids) found to be located in close proximity to *T. pallidum* miniproteins identified above, was performed using the protein structure modeling server, Phyre2^10^ (Kelley et al., 2015).

### Bioinformatics Pipeline: Tp0451a and Tp0749 AMPCCR Prediction

Two identified AMP candidates [Tp0451a (accession number WP_014342798) and Tp0749 (accession number WP_010882194)] were further analyzed using our in-house bioinformatics pipeline (Figure 1C). This analysis was performed to help confirm the initial AMP predictions generated by the three prediction servers and to identify potential antimicrobial peptide critical core regions (AMPCCRs) within Tp0451a and Tp0749. This pipeline was comprised of the following four stages:

(i) **Multi-server AMPCCR mapping**

Full-length amino acid sequences of Tp0451a and Tp0749 were submitted to four servers: AMPA^11^ (Torrent et al., 2012), CAMP antimicrobial region prediction server (CAMP-ARP)^12^ (Waghu et al., 2016), AntiBP^13^ (Lata et al., 2007), and AntiBP2^14^ (Lata et al., 2010). Potential AMPCCRs were identified in each protein based on high probability scoring regions (15–23 amino acid stretches) that were predicted by three or more servers.

(ii) **Structure analyses and modeling**

Secondary structure analyses of potential AMPCCRs were performed using the structure prediction servers Jpred 4^15^ (Drozdetskiy et al., 2015) and PSIPRED 4.0^16^ (Jones, 1999) and the alpha helix screening and physicochemical characterization server, HeliQuest^17^ (Gautier et al., 2008). Structure modeling of potential AMPCCRs based on PSIPRED secondary structure predictions were performed using the de novo peptide structure prediction server, PEP-FOLD 3^18^ (Thevenet et al., 2012; Shen et al., 2014) and Swiss Model^19^ (Bienert et al., 2017; Waterhouse et al., 2018). All models were then vetted through the comparative protein structure modelling server, Modeller^20^ (Webb and Sali, 2016) and the homology modeling program, ICM (Molsoft L.L.C., CA, United States)^21^ (Cardozo et al., 1995), for their lowest normalized discrete optimized protein energy value (zDOPE) and GA341 score closest to 1. Comparative homology modeling using structure-based alignment was performed using PROMALS3D^22^ (Pei et al., 2008). Together, these structure prediction analyses were used to support the multi-server AMPCCR predictions through the identification of structural folds known to be important for AMP function and for facilitating subsequent peptide design via the prediction of intact secondary structure elements.

(iii) **AMPCCR homology analyses**

Amino acid homology searches using the APD3 database (see text footnote 9) (Wang et al., 2016) were employed to determine similarity and identity of predicted *T. pallidum* AMPCCRs with known and experimentally-validated AMPs and for the identification of short orthologous AMP sequences that would be otherwise missed using the NCBI BLAST tools^23^ (Altschul et al., 1990);

(iv) **AMPCCR cell penetration prediction**

AMPCCR cell penetrating abilities, a key functional feature of AMPs, were predicted using the CellPPD Protein Scanning Tool^24^ (Gautam et al., 2013) (peptide fragment length = 10; prediction method = SVM based with scores ranging from –1.0 to +1.0). To increase prediction stringency, the SVM threshold for positive cell penetrating peptide predictions was increased from the default threshold (0) to ≥0.1.

### RNA Extraction and RT-PCR

RNA was isolated and purified from *in vivo*-harvested *T. pallidum* subsp. *pallidum* (Nichols strain) using Invitrogen TRIzol™ reagent (Thermo Fisher Scientific, MA United States) and the RNeasy mini kit (Qiagen, ON, Canada), according to the manufacturer’s instructions. RT-PCR was performed (after genomic DNA digestion/removal) using the orientation-specific RT-PCR sense (5’-aatgtcggctaccatcgctc) and antisense (5’-acgtgctctgccaattactgc) primers for *tp0451a* and the Invitrogen SuperScript™ IV First-Strand Synthesis System (Thermo Fisher Scientific), according to the manufacturer’s instructions. The negative control RT-PCR reaction did not include reverse transcriptase. PCR products were electrophoresed on agarose gels and visualized with ethidium bromide staining.

### Peptide Synthesis

For the experimental validation of antimicrobial and immunomodulatory activity, four putative AMPCCR peptides (Table 1) from two *T. pallidum* miniproteins that were identified using our AMP bioinformatics prediction pipeline (Tp0451a_N, Tp0451a_C, Tp0749_N, and Tp0749_C), a cysteine-to-serine substituted version of Tp0749_C (Tp0749_C_C61S), and a cysteine-to-serine substituted version of Tp0451a_C (Tp0451a_C_C85S), were synthesized without chemical modifications using the PepPower™ solid state peptide synthesis (SSPS) platform at GenScript (NJ, United States). The known AMP, human cathelicidin LL-37 (Turner et al., 1998), the known bullfrog (*Rana [Lithobates] catesbeiana*) AMP, RaCa-2 (Li et al., 2022), a scrambled version of LL-37 (sLL-37), and a peptide (Tp0751_p5) from the *T. pallidum* adhesin Tp0751 (Cameron et al., 2005) (Table 1) were also synthesized via the same SSPS platform at GenScript, and used as positive (LL-37 and RaCa-2) and negative (sLL-37 and Tp0751_p5) controls in antimicrobial susceptibility and immunomodulation assays, as described below.

**TABLE 1:** Chemically synthesized peptides used in the current study.

Peptide name	Peptide source and description	Amino acid sequence
Tp0451a_N	Tp AMPCCR (Tp0451a N-terminal peptide)	GCGSHCNCNVGYHRSLHCYGNELHGKQCGFSRCG
Tp0451a_C	Tp AMPCCR (Tp0451a C-terminal peptide)	IGRARAITHTWGIWCRWGKVWRRS
Tp0749_N	Tp AMPCCR (Tp0749 N-terminal peptide)	PFMQVITWARLYHKNQKRYEKIKK
Tp0749_C	Tp AMPCCR (Tp0749 C-terminal peptide)	KGIVAERILKPCVRRKVNGKFRS
Tp0451a_C_C85S	C-to-S substituted version of Tp0451a_C	IGRARAITHTWGIW**S**RWGKVWRRS
Tp0749_C_C61S	C-to-S substituted version of Tp0749_C	KGIVAERILKP**S**VRRKVNGKFRS
LL-37 (+ve)	Known human cathelicidin AMP	LLGDFFRKSKEKIGKEFKRIVQRIKDFLRNLVPRTES
RaCa-2 (+ve)	Known bullfrog AMP	FFPIIARLAAKVIPSLVCAVTKKC
sLL-37 (-ve)	Scrambled version of LL-37	RSLEGTDRFPFVRLKNSRKLEFKDIKGIKREQFVKIL
Tp0751_p5 (-ve)	*T. pallidum* peptide from adhesin Tp0751	AMRIALWNRATHGEQGALQHLLAG

*Tp, T. pallidum; AMPCCR, antimicrobial peptide critical core region (identified in the current study); -ve, negative control peptide; +ve, positive control peptide.*

### Antimicrobial Susceptibility Assay—Broth Microdilution

The broth microdilution technique (Wiegand et al., 2008) was used to determine if the *T. pallidum* peptides are capable of exhibiting bacteriostatic [minimal inhibitory concentration (MIC) measurements] and/or bactericidal [minimal bactericidal concentration (MBC) measurements] activity. Bacterial suspensions were prepared by transferring bacterial colonies into MHB and resuspending using a vortex mixer to ensure complete suspension of any bacterial aggregates. Turbidity of the colony suspensions was adjusted spectrophotometrically to the required optical densities to achieve a turbidity equivalent to that of a 0.5 McFarland standard (1–2 × 10^8^ CFU/mL) followed by dilution in MHB to achieve the standardized microbial inoculum of approximately 5 × 10^5^ CFU/mL. Total viable counts (TVC) were routinely performed on all inoculum suspensions to ensure correct bacterial cell densities. The standardized bacterial suspensions were then incubated with two-fold serial dilutions of each peptide (dissolved in 11 μL of ultrapure sterile water; final peptide concentration range of 256 μg/mL–0.5 μg/mL) in Greiner polypropylene round bottom 96-well microtiter plates (Sigma-Aldrich, MO, United States). Each peptide was tested once per experiment, with a range of 3–9 independent experiments performed per peptide. Negative growth/sterility control wells contained bacterial growth media (100 μL) and the peptide solvent (11 μL of ultrapure sterile water). Positive growth control wells contained the standardized number of bacterial cells (100 μL of ∼ 5.0 × 10^5^ CFU/mL) and peptide solvent (11 μL of ultrapure sterile water). Plates were incubated at 37°C for 16–24 h and MICs were determined using the unaided eye to identify the lowest concentration of AMP that inhibited visible growth of the tested bacterial species. If the sterility control well was turbid, the test was not considered valid. MBCs were determined by plating the entire content of the wells containing the peptide/bacteria mixture representing the MIC and the entire contents of the preceding wells containing two-fold and four-fold more concentrated AMP dilutions onto nonselective agar plates. Plates were incubated for 24 h at 37°C and MBCs were calculated as the percentage of bacteria killed at the different AMP concentrations tested (decrease in TVCs from the MBC plates compared to the initial bacterial suspension of ∼5 × 10^5^ CFU/mL).

### Antimicrobial Susceptibility Assay—*Neisseria* Modified Agar Dilution Method

The potential antimicrobial activity of the *T. pallidum* peptides against *N. gonorrhoeae* was determined using a modified agar dilution method. In this assay, *N. gonorrhoeae* colonies from GC chocolate agar plates were resuspended in MHB and the turbidity of the suspension was adjusted, as described above, to achieve the standardized microbial inoculum of approximately 5 × 10^5^ CFU/mL. A two-fold serial dilution of the peptides (11 μL in sterile ultrapure water) was then prepared in wells 1–10 of a 96-well sterile polypropylene plate to obtain a dilution series corresponding to 10 times the required testing concentrations (2,560, 1,280, 640, 320, 160, 80, 40, 20, 10, 5 μg/mL). The bacterial suspension (100 μL) was dispensed into the wells containing the peptides. A negative growth/sterility control (well 12) contained bacterial growth media (100 μL) and the peptide solvent (11 μL of ultrapure sterile water). A positive growth control (well 11) contained the standardized number of bacterial cells (100 μL of ∼ 5.0 × 10^5^ CFU/mL) and peptide solvent (11 μL of ultrapure sterile water). The 96-well plate was then incubated at 37°C in an atmosphere of 5% CO_2_ for 3 h to allow for peptide binding and antimicrobial activity. After the incubation period, an aliquot (20 μL) from wells 1–12 was removed and spotted onto the surface of a GC chocolate agar plate. *N. gonorrhoeae* spotted plates and TVC plates were incubated at 37°C in an atmosphere of 5% CO_2_ for 18–24 h. Following the incubation period, MICs were determined by identifying the lowest concentration of peptide that completely inhibited visible growth on the agar plate. To determine the bactericidal activity of peptides, total viable counts (TVCs) were also prepared on GC chocolate agar plates for the 3 h-incubated peptide/bacteria mixtures. These counts were compared with TVCs from the corresponding positive growth wells to give the percentage of bacteria killed by each of the peptides following the 3-h incubation.

### Antimicrobial Susceptibility Assay – *T. pallidum*

An antimicrobial susceptibility assay was developed to assess the activity of the four treponemal peptides (Tp0451a_N, Tp0451a_C, Tp0749_N, and Tp0749_C), an equimolar mix of Tp0451a_N and Tp0451a_C, and the negative (Tp0751_p5) and positive (LL-37) control peptides, against *T. pallidum*. *In vitro*-grown *T. pallidum* (100μL; 1.0–1.2 × 10^6^ Tp/mL), prepared as described above, were incubated with each peptide at three concentrations (4, 16, 64 μg/mL) or the Tp0451a_N/Tp0451a_C mix (21.6 μM; ∼85 μg/mL Tp0451a_N and ∼64 μg/mL Tp0451a_C) at 34°C in an atmosphere of 93.5% nitrogen, 5% carbon dioxide, and 1.5% oxygen. Darkfield microscopy was used to monitor *T. pallidum* viability by counting motile treponemes at 0, 1, 2, and 4 h post co-incubation. For each viability measurement, at least 50 treponemes were observed for each sample at each time point.

### THP-1 Monocyte Culture and Macrophage-Like Differentiation

Human THP-1 (ATCC TIB-202) monocytes (American Type Culture Collection, VA, United States) were cultured and maintained in 5% CO_2_ at 37°C in RPMI-1640 medium (Gibco, Life Technologies, ON, Canada) supplemented with 10% (v/v) fetal bovine serum (FBS) (Fisher Scientific, ON, Canada), 0.05 mM 2-mercaptoethanol (BME) (Sigma-Aldrich, ON, Canada), penicillin (100 units), and streptomycin (0.1 mg/mL) (Sigma-Aldrich, ON, Canada) (hereafter referred to as “complete RPMI-1640 medium”). Cells were passaged at a density of 8 × 10^5^ cells/mL to a maximum of 15 passages and re-seeded at 3 × 10^5^ cells/mL for maintenance. For differentiation into plastic-adherent macrophage-like cells, THP-1 monocytes were seeded at a density of 3 × 10^5^ cells/mL in T75 tissue culture flasks (Fisher Scientific, Ottawa, ON, Canada) and stimulated with a low concentration (25 ng/mL) of phorbol-12-myristate-13-acetate (PMA) (Sigma-Aldrich, ON, Canada) in complete RPMI-1640 medium for 48 h in 5% CO_2_ at 37°C. The PMA-mediated differentiation method results in the generation of cells with phenotypic characteristics that are similar to human peripheral blood mononuclear cell (PBMC) monocyte-derived macrophages; they are adherent, larger, more phagocytic, are less proliferative, and exhibit cell surface markers that are characteristic of macrophages (Chanput et al., 2014).

Following incubation with PMA, light microscopy was used to ensure the differentiated cells were adherent, and exhibited morphological changes consistent with PBMC monocyte-derived macrophages. Non-adherent cells were then removed by washing with sterile, calcium- and magnesium-free phosphate-buffered saline (PBS) (ThermoFisher Scientific, MA United States). Plastic-adherent macrophage-like cells were detached by a three- to five-minute treatment with trypsin-EDTA (0.05%) (ThermoFisher Scientific, MA, United States) and physical agitation. The macrophage-like cells were then centrifuged at 1,500 rpm using a Sorvall Model STR04 centrifuge (ThermoFisher Scientific, MA United States) for 5 min at room temperature and seeded in PMA-free complete RPMI-1640 media, as described below.

### AMP Stimulatory and AMP/IL-32γ Co-stimulatory Immunomodulation Assays

THP-1 monocytes and THP-1 macrophage-like cells were seeded into the wells of 12-well plates (Thermo Fisher Scientific, ON, Canada) in complete RPMI-1640 medium (1 mL) at a density of 0.5 × 10^6^ cells/mL. Following seeding and prior to stimulation, macrophage-like cells were rested overnight. For AMP alone stimulation, monocytes or macrophages were exposed for 24 h at 37°C in 5% CO_2_ to either (i) no stimulation (negative control for baseline cytokine production), (ii) lipopolysaccharide [LPS; from *S. enterica* serovar Typhimurium (Sigma-Aldrich, ON, Canada)] (positive control for cytokine production; final concentration 1.0 μg/mL), or (iii) the test peptides (control peptides and potential *T. pallidum* AMPs listed in Table 1; final concentration 25 μg/mL). For AMP/IL-32γ co-stimulation, 20 ng/mL IL-32γ (R&D Systems, MN, United States) in fresh complete RPMI-1640 medium was added to the rested macrophages. IL-32γ stimulation was immediately followed by co-stimulation by the addition of the test peptides at a final concentration of 25 μg/mL. Macrophage cells left unstimulated or stimulated with IL-32γ alone were used as negative and positive controls, respectively. Cells were stimulated for 24 h at 37°C in 5% CO_2_. Following stimulation, monocytes and macrophage cells were centrifuged at 1,500 rpm using a Sorvall Model STR04 centrifuge (ThermoFisher Scientific, MA, United States) for 5 min at room temperature and the cell-free supernatant was stored at -80°C prior to quantification of cytokine levels, as described below.

### THP-1 Monocyte and Macrophage Cytokine Expression Analyses

The BD™ Cytometric Bead Array (CBA) system (BD Biosciences, CA, United States) was used to quantify the expression of tumor necrosis factor (TNF), MCP-1, IL-6, IL-8, IL-10, and IL-1β according to manufacturer’s instructions. For statistical analyses, data were analyzed for normality using a D’Agostino-Pearson omnibus normality test and a Shapiro–Wilk normality test. An ordinary one-way ANOVA followed by Dunnett’s multiple comparisons test was used to assess differences between three or more groups of normally distributed data. A Kruskal–Wallis test followed by Dunn’s multiple comparisons test was used to assess differences between three or more groups of data that were not normally distributed.

## Results

### Identification of *T. pallidum* Miniproteins of Unknown Function

Although the size of AMPs can vary greatly, ranging from approximately five amino acids to several hundred amino acids, a search of the AMP database, APD3^25^ (Wang et al., 2016), indicated that 97% of the 3324 AMPs listed at the time of analysis fall within the 10–150 amino acid size range. With this knowledge, we sought to identify potential *T. pallidum* AMPs by manually searching the whole proteome of *T. pallidum* and filtering for proteins containing 150 amino acids or less. This resulted in the identification of 151 miniproteins (≤150 amino acids) (Supplementary Table 1), representing ∼16% of the *T. pallidum* proteome. We then filtered for miniproteins with no assigned function or weak/incomplete annotated functions and for miniproteins with a potential AMP-related function, resulting in the identification of 68 proteins (Supplementary Table 2), representing 7% of the *T. pallidum* proteome. Genes corresponding to four of these 68 proteins (Tp0039, Tp0130, Tp0451a, and Tp0867) were not included in the latest annotation of the *T. pallidum* proteome (Nichols strain NC_021490), however, all four genes have been shown to be expressed at the transcript level (Smajs et al., 2005 and current study) justifying their inclusion in this study (Supplementary Table 2). Sixty-seven of the 68 miniproteins of unknown function that were identified in *T. pallidum* were annotated in the published proteome from July 2021 as either “hypothetical proteins,” “DUF (Domains of Unknown Function) domain-containing proteins,” or as proteins with motifs/domains that do not provide enough insight to confidently indicate potential protein functions (e.g., helix-turn-helix domain-containing proteins, DNA- or RNA-binding proteins, zinc ribbon domain-containing protein) (Supplementary Table 2). One of the 68 miniproteins was annotated as a putative CPBP family intramembrane metalloprotease (TPANIC_RS05485), some of which may be involved in AMP processing (Pei et al., 2011). However, this 91-amino acid treponemal protein is at least two-four-fold smaller than other bacterial CPBP proteins, is only predicted to contain one transmembrane segment (unlike the four or more present in known CPBP proteins), and does not contain the four conserved sequence motifs required for proteolytic activity that are found in other CPBP proteins (Pei et al., 2011). In light of these findings, Tp_RS05485 was included in the list of 68 miniproteins for further bioinformatics analyses (Supplementary Table 2).

### Prediction Analyses for the Identification and Ranking of Putative *T. pallidum* AMPs

**FIGURE 2:**
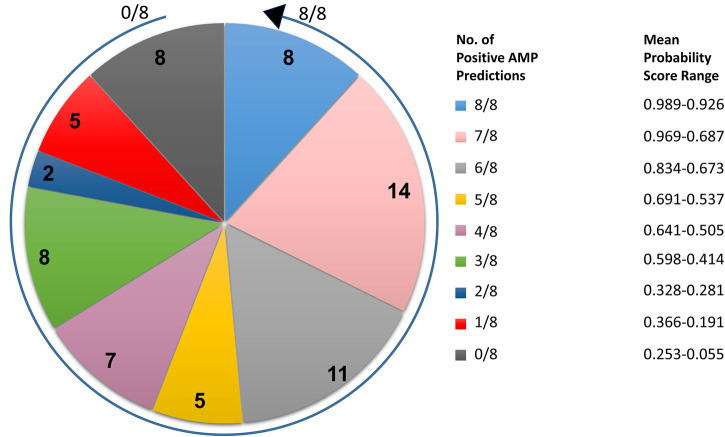
Distribution of the number of positive AMP predictions and corresponding mean probability score ranges for *T. pallidum* miniproteins of unknown function. Pie chart indicating the number of positive AMP predictions following submission of the full-length amino acid sequences of 68 *T. pallidum* miniproteins (≤150 amino acids) to three AMP prediction servers (eight AMP prediction algorithms total). The arrow begins at the 0/8 positive AMP prediction class and finishes at the 8/8 positive AMP prediction class. The corresponding mean probability score range for all proteins from each positive AMP prediction class is also shown.

Full-length amino acid sequences of all 68 miniproteins identified in the analyses described above were submitted to three AMP prediction servers. Prediction data were then used to rank the 68 treponemal proteins from most likely AMP (ranking 1/68) to least likely AMP (68/68) depending on (i) how many of the eight server algorithms produced positive AMP predictions and (ii) the mean probability scores from each of the AMP predictions for each protein. In summary, 45 high-priority *T. pallidum* AMP candidates predicted by at least four of the eight algorithms were assigned mean probability scores of at least 0.505 (50.5% probability) (Figure 2 and Supplementary Table 3). AMP prediction results for all 68 *T. pallidum* miniproteins and corresponding probability scores from each of the AMP prediction servers are listed in Supplementary Table 3.

### Leader Peptide Analyses of *T. pallidum* Miniproteins

Many bacterial AMPs are synthesized as inactive preproteins with N-terminal leader/signal peptides whose presence and cleavage are required for export and activation, respectively. These include signal peptides recognized by the general secretory (Sec) pathway [Sec-dependent signal peptides (Sec/SP1 peptides)] (Leer et al., 1995; Chiorean et al., 2018) and Sec-independent double-glycine/glycine-alanine (GG/GA) leader peptides that have been documented in AMPs from both Gram-positive and Gram-negative bacteria (Havarstein et al., 1994; Oman and van der Donk, 2010). The signal peptide prediction servers, SignalP and LipoP, were used to search for the presence of Sec-dependent SP1 signal peptides, which predicted the presence of Sec/SP1 signal peptides in only four of the 68 miniproteins (Supplementary Table 4). Manual searches of the 68 *T. pallidum* AMP candidates for the conserved double-glycine/glycine-alanine leader peptide motif identified 24 proteins that contain Glycine-Glycine and/or Glycine-Alanine pairs within the first 31 residues of the N-terminus (Supplementary Figure 1). WebLogo analysis of the 24 proteins identified an N-terminal region with similarity to the double-glycine/glycine-alanine leader peptide motif from AMPs from Gram-negative bacteria, suggesting the presence of a similar secretion/activation recognition signal in *T. pallidum* candidate AMPs (Supplementary Figure 2).

### Physicochemical Analyses of *T. pallidum* Miniproteins

**FIGURE 3:**
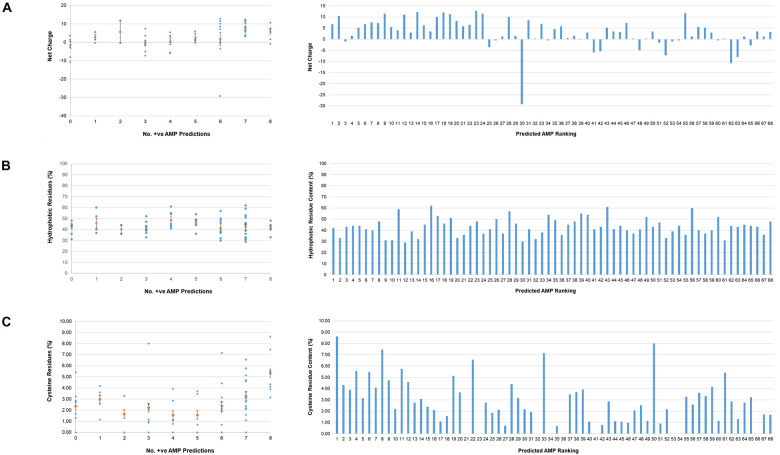
Physicochemical properties of *T. pallidum* miniproteins of unknown function. Physicochemical properties known to be important for AMP function were calculated for the 68 *T. pallidum* proteins (≤150 amino acids) of unknown function using the APD3 online AMP calculator and predictor tool. Left: Scatter plots depicting **(A)** net charges, **(B)** hydrophobic residue content, and **(C)** cysteine residue content (orange circles represent the mean values, +/- standard error) of all proteins from each positive AMP prediction class. Right: Bar graphs showing **(A)** net charge, **(B)** hydrophobic amino acid content, and **(C)** cysteine residue content of all 68 miniproteins (≤150 amino acids) from predicted AMP ranking 1–68. No. +ve AMP Predictions equates to the number of AMP prediction programs that assigned the miniprotein as an AMP.

Physicochemical properties known to be important for AMP function were calculated using the APD3 online AMP calculator and predictor tool. Consistent with the high content of arginine and lysine residues in AMPs, the top 22-ranking potential *T. pallidum* AMPs were found to have mean net charges at pH 7.0 of 5.56 (8 proteins with 8/8 positive AMP predictions) and 7.91 (14 proteins with 7/8 positive AMP predictions) (Figure 3A and Supplementary Table 4). Although no trend was observed between AMP likelihood rankings and the percentage of hydrophobic amino acids found within this group of proteins, the mean hydrophobic residue content of all 68 miniproteins was high (43.1%), with 66/68 proteins comprised of more than 30% hydrophobic residues (Figure 3B and Supplementary Table 4). Also observed was a trend in AMP likelihood rankings and the number / percentage of cysteine residues per protein. In general, cysteines were found to be more common in higher-ranking predicted AMPs (Figure 3C and Supplementary Table 4). In comparison to an average cysteine content of ∼1.9% found in all *T. pallidum* proteins [calculated from the Nichols strain (NC_021490) proteome], the top eight-ranking predicted AMPs (8/8 positive AMP predictions) contained on average almost three-fold more cysteines (mean cysteine residue content = 5.31%). Interestingly, the cysteine-rich nature of these *T. pallidum* miniproteins is shared with distinct classes of eukaryotic (Simmaco et al., 1994; Fahrner et al., 1996; Shafee et al., 2016) and prokaryotic (Baindara et al., 2017; Sugrue et al., 2020) AMPs, and thus may represent an important physicochemical property for protein structure and/or function.

### Expression Analyses of *T. pallidum* Miniproteins

Most of the 68 *T. pallidum* miniproteins from the current study are annotated in the published *T. pallidum* proteome as “hypothetical” proteins. However, DNA microarray-based analysis of the *T. pallidum* transcriptome following experimental rabbit infection (Smajs et al., 2005) demonstrated that 56/68 genes encoding these functionally uncharacterized proteins are expressed at the transcriptional level (Supplementary Table 5). Transcripts from most of the other 12 genes were not searched for in the study (Smajs et al., 2005) as they were not annotated in the *T. pallidum* genome at the time the study was performed. In addition, peptides from 15/68 miniproteins were detected in mass spectrometry-based proteomics studies of rabbit infections (McGill et al., 2010; Osbak et al., 2016), including the protein with the highest level of expression in the Osbak and colleagues study, Tp0214 (Supplementary Table 5). The use of trypsin for *T. pallidum* protein digestion in the two mass spectrometry studies may have contributed to the low number of miniproteins detected in these experiments. Given that miniproteins are small and contain high numbers of lysine and arginine residues, trypsin treatment, which results in cleavage after lysine and arginine residues, would be expected to cleave the miniproteins into many small peptides. Many of these peptides would be below the size detection limit, a major limiting factor for protein identification in mass spectrometry studies.

### Proteome Clustering of *T. pallidum* Miniproteins

**FIGURE 4:**
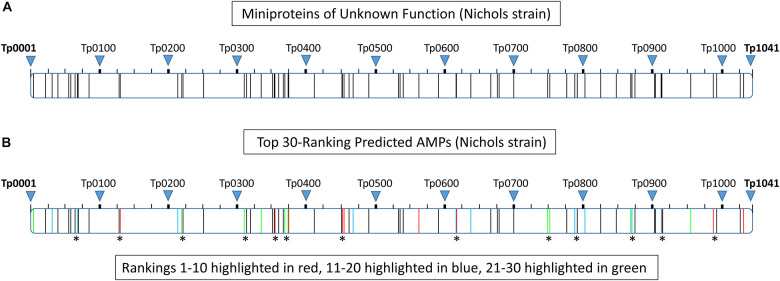
Proteome clustering of *T. pallidum* (Nichols strain) miniproteins of unknown function. Each of the *T. pallidum* (Nichols strain) proteins (≤150 amino acids) of unknown function were arranged from the lowest (Tp0004) to the highest (Tp1032) locus tag number within the proteome (Tp0001–Tp1041) and clusters comprised of at least two miniproteins separated by five or less intervening proteins were identified. **(A)** Schematic depicting the spatial arrangement of all miniproteins (≤50 amino acids) of unknown function within the *T. pallidum* (Nichols strain) proteome. The location of each protein within the proteome is represented by a vertical black line. **(B)** Schematic showing the spatial arrangement of the 30 top-ranking predicted AMPs within the *T. pallidum* (Nichols strain) proteome. The location of proteins corresponding to rankings 1–10, 11–20, and 21–30 are shown in red, blue, and green, respectively. Asterisks denote the location of 20 of the top 30-ranking predicted AMPs that are found to be located within 13 miniprotein clusters.

Bacterial genomic analyses show genes with related functions tend to form gene clusters (Tamames et al., 1997). To determine the spatial arrangement of all 68 miniproteins (≤150 amino acids) of unknown function within the *T. pallidum* proteome, each protein was arranged from the lowest (Tp0004) to the highest (Tp1032) locus tag number and clusters comprised of at least two miniproteins separated by five or less intervening proteins were identified. Forty-three of the 68 miniproteins (63%) were found to be located within one of 17 clusters, with 23 of the 43 proteins located in clusters comprised of at least three miniproteins of unknown function (Supplementary Table 6 and Figure 4A). Twenty of the top 30-ranking predicted AMPs (67%) were found to be located within 13 miniprotein clusters, with 11 of the 20 proteins located in clusters containing at least three miniproteins of unknown function (Supplementary Table 6 and Figure 4B). The top-ranking predicted AMP (Tp_RS02215) was found in a three-miniprotein cluster including the 8*^th^*-ranking predicted AMP (Tp0451a). Interestingly, analysis of the surrounding proteome identified two annotated proteins of note including the outer-membrane inner-leaflet-associated lipoprotein, Tp0453, which contains multiple outer membrane-inserting amphipathic alpha helices that result in membrane bilayer destabilization and enhanced permeability (Hazlett et al., 2005; Luthra et al., 2011). Also included in this region is Tp0454; structure modeling of Tp0454 using Phyre2 predicted tertiary structure similarity to several response regulators (Supplementary Table 6), including the DNA-binding response regulator, PhoP, from the PhoP-PhoQ two-component system that is a central regulator for AMP resistance in Gram-negative bacteria (Brodsky and Gunn, 2005). Proteome functional annotation analyses and Phyre2 structure modeling of open reading frames located close to other putative AMPs identified several additional potential homologs and structural orthologs with potential functions that are consistent with AMP secretion, activation, transport, and self-immunity (Supplementary Table 6), including the ORFs Tp0405 and Tp0688 that have been previously annotated as self-immunity proteins (Fraser et al., 1998). The close spatial arrangement of the miniproteins, in particular the high-ranking predicted AMPs, together with the observed proximity of potential AMP accessory proteins in the proteome of *T. pallidum* is consistent with the concept that functionally-related genes have a tendency to form clusters within bacterial genomes (Tamames et al., 1997).

### AMP Candidate Selection: Tp0451a and Tp0749

Two AMP candidates, Tp0451a (accession number WP_014342798) and Tp0749 (accession number WP_010882194), were selected for the identification of potential AMPCCRs, the important minimalistic functional regions of AMPs. Tp0451a was selected as it (i) is one of the top-ranking predicted AMPs (8/8 positive AMP predictions, mean probability score of 92.6%) (Supplementary Table 3), (ii) possesses classical AMP properties (high content of positively-charged and hydrophobic amino acid residues) (Supplementary Table 4), (iii) is clustered in the proteome with several other potential AMPs/related proteins, as described above, and (iv) *tp0451a* is expressed at the transcript level, as described below. Although Tp0749 is a lower-ranking predicted AMP (ranked 24/68, 6/8 positive AMP predictions, mean probability score of 78.4%) (Supplementary Table 3), it was selected for further analyses as it (i) is highly positively-charged and contains high hydrophobic content, consistent with pore-forming AMPs (Supplementary Table 4), (ii) was identified in preliminary bioinformatics analyses as having clearly defined potential critical core regions, indicative of future success in AMPCCR design and synthesis, (iii) is known to be expressed at the transcript level (Smajs et al., 2005), unlike several of the higher-ranking predicted AMPs (Supplementary Table 5), (iv) is the second highest expressed ORF at the transcript level in the top-30 ranking *T. pallidum* miniproteins (Smajs et al., 2005) (Supplementary Table 5), and (v) of particular importance, it is one of only six minproteins within the top-30 ranking predicted AMPs whose expression has been detected at the protein level in experimental rabbit infections (Osbak et al., 2016) (Supplementary Table 5). To date, protein expression of all eight miniproteins from the top-eight ranking predicted AMPs (8/8 positive AMP predictions, mean probability score range 98.9–92.6%) has not been demonstrated in rabbit models of infection (McGill et al., 2010; Osbak et al., 2016), and only three of the top-eight ranking predicted AMPs have been shown to be expressed at the transcript level (Smajs et al., 2005). The strong experimental evidence confirming expression of Tp0749 at both the RNA and protein levels in rabbit infections increased the prioritization of this predicted treponemal AMP over higher-ranking predicted AMPs for further bioinformatics and functional characterization studies.

### Reverse Transcription-PCR Analysis of *tp0451a*

To confirm expression of *tp0451a*, we analyzed RNA isolated from *T. pallidum* by reverse transcription PCR (RT-PCR) using sense and antisense primers. When reverse transcriptase was present (RT+), the primer pair amplified a 198 base pair product, matching a similarly sized amplicon generated from *T. pallidum* genomic DNA (Supplementary Figure 3 lanes 2 and 4, respectively). In comparison, only a very faint amplicon was detected when reverse transcriptase was omitted from the RT-PCR reaction (RT-) (Supplementary Figure 3 lane 3), indicating that the 198 base pair product from the RT+ reaction was amplified from RNA and not contaminating DNA. Together with the previous finding that showed expression of Tp0749 at the protein level (Osbak et al., 2016), this result allowed us to proceed with investigations into potential AMP activity in *T. pallidum* by focusing on two miniproteins, Tp0451a and Tp0749, that are known to be expressed at either the transcript or protein level.

### Bioinformatic Identification of Potential Critical Core Regions in Two Putative *T. pallidum* AMPs

**FIGURE 5:**
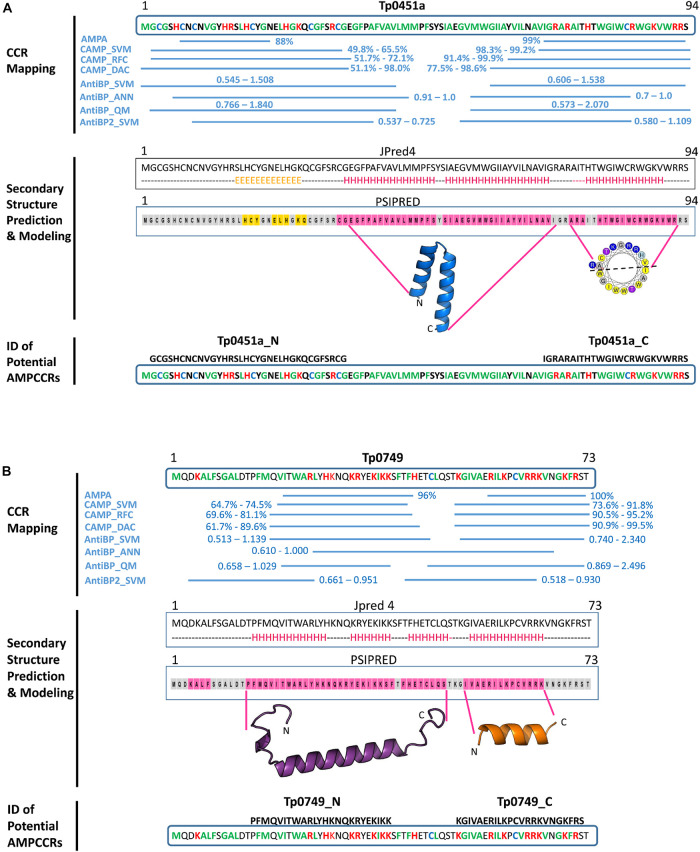
*In silico* identification of potential *T. pallidum* AMPCCRs. The critical core regions of two candidate AMPs, **(A)** Tp0451a and **(B)** Tp0749, were predicted using our bioinformatics pipeline. The first step of the pipeline involved CCR mapping **(A,B**, top): four prediction servers [AMPA (one algorithm), CAMP (three algorithms), AntiBP (three algorithms), and AntiBP2 (one algorithm)] were used to identify the amino acid boundaries of potential antimicrobial active regions (critical core regions, CCRs). High probability/scoring regions predicted by at least three of the four servers are shown with their corresponding probabilities (AMPA and CAMP algorithms) or scores (AntiBP and AntiBP2 algorithms). Hydrophobic residues: green; Positively-charged residues: red; Cysteines: blue. In the second step of the pipeline, secondary structure analyses and modeling were performed (**A** and **B** panels, middle): secondary structure analyses of the full-length proteins were performed using Jpred 4 (H: alpha helix; E: beta strand; dashed line: coiled) and PSIPRED (pink highlight: alpha helix; orange highlight: beta strand; gray highlight: coiled). HeliQuest was used to generate helical wheel diagrams for potential alpha helices (yellow: hydrophobic residues; purple: serine or threonine; blue: positively charged residues: gray: glycine or alanine). Structure modeling using Modeller generated a confident model for the central region of Tp0451a (residues E36-I71), but confident models were not generated for the N- or C-terminal regions. Structure modeling using a combination of PEP-FOLD-2, Swiss-Model, Molsoft ICM, and Modeller generated models for the N- and C-terminal regions of Tp0749 (residues P14-S48 and I52-K65, respectively). Together, these findings allowed for the identification of two potential critical core regions within the N-terminus (Tp0451a_N and Tp0749_N) and C-terminus (Tp0451a_C and Tp0749_C) of Tp0451a and Tp0749 (**A** and **B** panels, bottom).

The first step for mapping AMPCCRs in Tp0451a and Tp0749 involved using four prediction servers [AMPA (one algorithm), CAMP (three algorithms), AntiBP (three algorithms), and AntiBP2 (one algorithm)] to identify the amino acid boundaries of potential antimicrobial active regions (critical core regions, CCRs) within the two *T. pallidum* proteins based on clusters of high probability scoring regions predicted by at least three of the four servers. For both Tp0451a and Tp0749, two potential active regions were identified in the N-and C-termini of each protein (Figures 5A,B).

In the second step of our pipeline, secondary structure analyses and structure modeling of the two proteins and their identified potential active regions were performed. For Tp0451a, Jpred 4, and PSIPRED analyses predicted a predominantly coiled/beta strand structure that corresponded to the N-terminal predicted active region and an alpha helical structure that corresponded to the C-terminal predicted active region (Figure 5A). HeliQuest analysis showed that the predicted C-terminal alpha helix exhibited amphipathic properties (Figure 5A), a common structural characteristic in AMPs that is important for membrane integration and pore formation. Structure modeling using a combination of PEP-FOLD 2, Swiss Model, Modeller and Molsoft ICM was unable to generate high confidence models for either the N- or C-terminal regions that correspond to the predicted antimicrobial active regions. However, a robust model was generated for the intervening central region (residues E36-I71) (Figure 5A and Supplementary Figure 4). In agreement with the secondary structure predictions, this central region was modeled as two hydrophobic alpha helices. The structure prediction analyses were consistent with the multi-server AMPCCR mapping predictions by defining potential structural elements, one of which is important for AMP function, that corresponded to high-scoring predicted active regions. Together, these findings allowed for the identification of two potential critical core regions within the N- (Tp0451a_N) and C-terminus (Tp0451a_C) of Tp0451a (Figure 5A).

**FIGURE 6:**
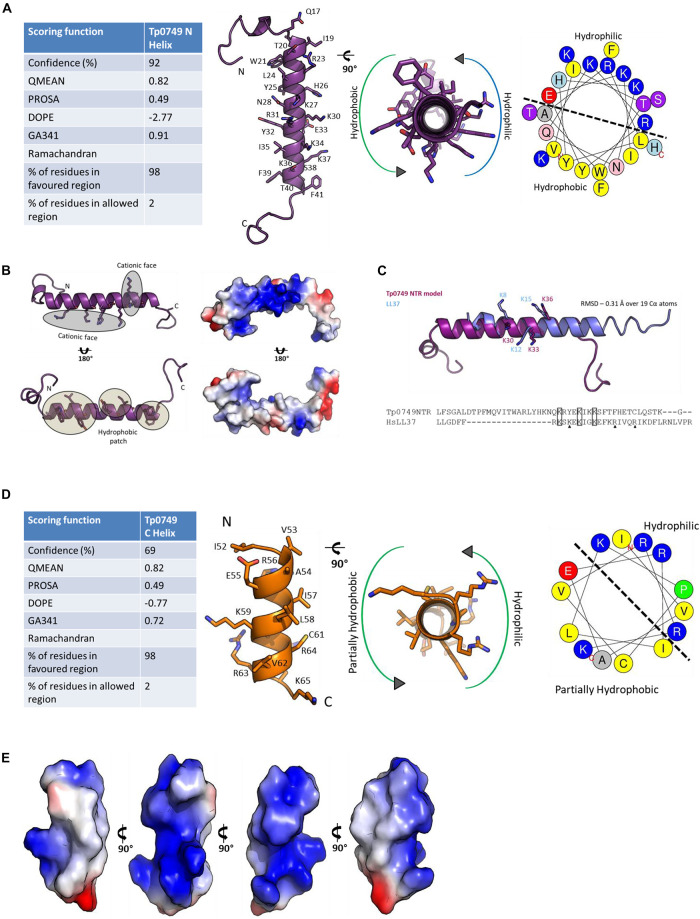
Structure modeling of the candidate *T. pallidum* AMP, Tp0749. **(A**, Left): Table showing the scoring functions of the Tp0749 N-terminal model (residues P14-S48) generated by a combination of PEP-FOLD-2, Swiss-Model, Molsoft ICM, and Modeller. (Middle) Model ribbon structure of Tp0749 residues P14-S48 and rotated view showing amphipathicity. (Right) Helical wheel schematic of Tp0749 (P14-S48) generated using HeliQuest. Dashed line separates the hydrophilic/polar and hydrophobic/non-polar faces of the predicted alpha helix. **(B)** Ribbon and surface/charge distribution images of the Tp0749 (P14-S48) model showing one face of the alpha helix rich in positively-charged/polar residues (blue) and the opposing face rich in hydrophobic/non-polar residues (white). Red: negatively-charged/polar residues. **(C)** PROMALS3D was used to generate a structure-based comparative model of the Tp0749 N-terminal alpha helix model (P14-S48) using the structure of the known AMP, human cathelicidin LL-37 (PDB:5NMN) as a template. RMSD—0.31 Å over 19 Cα atoms and 3/6 positively charged residues involved in binding target cell membrane lipids are conserved (indicated by rectangles; triangles show non-conserved residues). **(D)** Left: Table showing the scoring functions of the Tp0749 C-terminal model (residues I52-K65) generated by using a combination of PEP-FOLD-2, Swiss-Model, Molsoft ICM, and Modeller. (Middle) Model ribbon structure of Tp0749 residues I52-K65 and rotated view showing partial amphipathicity (red: negatively charged/polar residue side chains; blue: positively charged/polar residue side chains; yellow: cysteine residue side chain). (Right) Helical wheel schematic of Tp0749 (I52-K65) generated using HeliQuest. Dashed line separates the hydrophilic/polar and partially hydrophobic/non-polar faces of the predicted alpha helix. **(E)** Surface and charge distribution views of Tp0749 (I52-K65) (red: negatively charged/polar residues; blue: positively charged/polar residues; white: hydrophobic/non-polar residues).

For Tp0749, both Jpred 4 and PSIPRED analyses predicted alpha helices that corresponded to the N- and C-terminal predicted active regions (Figure 5B). Consistently, a high confidence (92%) alpha helix was modeled for the N-terminal region that was predicted to exhibit antimicrobial activity (Figures 5B, 6A). Importantly for potential AMP function, this modeled N-terminal alpha helix was also shown to be amphipathic with one face of the helix rich in positively-charged residues and the opposing face rich in hydrophobic residues (Figures 6A,B). In addition, a structure-based alignment using PROMALS3D of the Tp0749 N-terminal alpha helix model and a solved structure from the known AMP, human cathelicidin LL-37 (PDB:5NMN), predicted structural similarity between the two peptides (RMSD—0.31 Å over 19 Cα atoms) and conservation of 3/6 positively charged residues involved in binding target cell membrane lipids (Sancho-Vaello et al., 2017) (Figure 6C). In agreement with secondary structure predictions, a lower confidence (69%) partially amphipathic alpha helix model was also modeled for the C-terminal predicted antimicrobial active region (Figures 5B, 6D,E). The combined approach of multi-server AMPCCR mapping, secondary structure prediction, and modeling allowed for the identification of two potential critical core regions located in the N- (Tp0749_N) and C- (Tp0749_C) terminus of Tp0749 (Figure 5B).

To help further resolve the potential active regions identified above, the four candidate AMPCCRs were then analyzed for similarity with known, experimentally-validated AMPs from the APD3 database and for their predicted cell penetrating capabilities using CellPPD. Amino acid sequence-based homology searches identified similarities of each of the four AMPCCRs with established AMPs with homologies ranging between 37 and 44% (Table 2). CellPPD analysis also predicted that three of the four potential AMPCCRs with predicted amphipathic alpha helices (Tp0451a_C, Tp0749_N, and Tp0749_C) (Figures 5A, B, 6) contain peptide stretches that may have the ability to penetrate cell membranes (Table 3), a key functional feature of AMPs. The positive control peptide (LL-37) was also predicted to contain cell penetrating peptide regions, unlike the negative control peptide, Tp0751_p5 (Table 3). Together, these results bolstered the initial AMP predictions generated by the four prediction servers and identified two potential AMPCCRs within each of Tp0451a and Tp0749. These putative functionally-active core regions were prioritized for peptide synthesis and AMP functional characterization studies.

**TABLE 2:** Known AMPs with the highest similarity to four *T. pallidum* candidate AMPCCRs.

*Tp* AMPCCR	Similar known AMP	AA Similarity (%)	Source	Activity
Tp0451a_N	Beta Defensin 6 (Yamaguchi et al., 2001)	37.20	Mammals	Anti-Gram-negative
Tp0451a_C	ecPis3 (Zhuang et al., 2017)	37.03	Fish	Anti-Gram-negative Anti-Gram-positive Antifungal Antiparasitic
Tp0749_N	Brevinin-1CHb (Conlon et al., 2011)	37.03	Amphibians	Anti-Gram-negative Anti-Gram-positive Antifungal
Tp0749_C	P15s (Oyama et al., 2017)	44.44	Mammals	Anti-Gram-negative

*The four candidate AMPCCRs from T. pallidum were analyzed for amino acid sequence similarity with established, experimentally-validated AMPs using homology searches in the APD3 database. Tp, Treponema pallidum; AA, amino acid.*

**TABLE 3:** *Treponema pallidum* candidate AMPCCRs with predicted cell penetrating abilities.

*Tp* AMPCCR	Cell penetrating predictions	SVM Score
Tp0451a_N	GCGSHCNCNVGYHRSLHCYGNELHGKQCGFSRCG	Non-CPP
Tp0451a_C	IGRARAITHTWGI**WCRWGKVWRR**S	0.38
Tp0451a_C	IGRARAITHTW**GIWCRWGKVW**RRS	0.12
Tp0749_N	PFMQVITWARLYH**KNQKRYEKIK**K	0.36
Tp0749_N	PFMQVITWARLYHK**NQKRYEKIKK**	0.33
Tp0749_N	PFMQ**VITWARLYHK**NQKRYEKIKK	0.10
Tp0749_C	KGIVAE**RILKPCVRRK**VNGKFRS	0.46
Tp0749_C	**KGIVAERILK**PCVRRKVNGKFRS	0.21
Tp0749_C	KGIVAERILKPC**VRRKVNGKFR**S	0.20
Tp0749_C	KGIVAERILK**PCVRRKVNGK**FRS	0.19
Tp0749_C	KGIV**AERILKPCVR**RKVNGKFRS	0.15
LL-37 (+ve)	LLGDF**FRKSKEKIGK**EFKRIVQRIKDFLRNLVPRTES	0.31
LL-37 (+ve)	LL**GDFFRKSKEK**IGKEFKRIVQRIKDFLRNLVPRTES	0.16
LL-37 (+ve)	LLGDFFRKSKEKIGKEFK**RIVQRIKDFL**RNLVPRTES	0.15
LL-37 (+ve)	LLGDFFRKSKEKIGKEF**KRIVQRIKDF**LRNLVPRTES	0.13
LL-37 (+ve)	LLGDFFRKSKEKIGKE**FKRIVQRIKD**FLRNLVPRTES	0.11
LL-37 (+ve)	LLGDFFRKSKEKIG**KEFKRIVQRI**KDFLRNLVPRTES	0.10
Tp0751_p5 (–ve)	AMRIALWNRATHGEQGALQHLLAG	Non-CPP

*The four candidate AMPCCRs from T. pallidum and positive (LL-37) and negative (Tp0751_p5) control peptides were analyzed for predicted cell penetrating capabilities using CellPPD (10 amino acid peptide scan). Predicted 10-amino acid cell penetrating peptides are highlighted in bold. Corresponding Support Vector Machine (SVM) scores [0.1 (lowest probability) – 1.0 (highest probability)] for each predicted cell penetrating peptide (in bold) are shown. Tp, Treponema pallidum; non-CPP, no cell penetrating peptides predicted.*

### *In vitro* Antimicrobial Activity of *T. pallidum* AMPCCR Candidates

To evaluate the potential antimicrobial activity of the four predicted *T. pallidum* AMPCCRs identified via our bioinformatics pipeline, synthetic peptides were produced and antimicrobial susceptibility assays were performed to test for bacteriostatic and bactericidal activities against a panel of biologically and clinically relevant Gram-negative and Gram-positive bacteria. In broth microdilution assays, all four candidate AMPCCRs were active against *M. smegmatis* (Table 4). Consistent with our bioinformatics pipeline analyses, the three *T. pallidum* peptides with predicted amphipathic alpha helices (Tp0451a_C, Tp0749_N, and Tp0749_C) (Figures 5A,B, 6) and highest AMPCCR mapping scores (Figures 5A,B) all exhibited robust anti-mycobacterial activity, unlike the lower-scoring Tp0451a_N. The lack of activity in the latter peptide aligns with the observation that this was the only peptide that lacked predicted (amphipathic) helical structure (Figure 5A). With the exception of Tp0451a_N, the treponemal peptides exhibited anti-mycobacterial activity that was similar to the positive control AMPs, LL-37 and RaCa-2. Tp0451a_N showed no antimicrobial activity against any of the other tested bacteria, whereas Tp0451a_C showed considerable bacteriostatic and bactericidal potency toward the Gram-positive bacterium, *S. pyogenes*, to a level that exceeded that observed with the positive control AMPs, LL-37 and RaCa-2. None of the other three treponemal peptides exhibited anti-streptococcal activity and all four treponemal peptides were inactive against *S. enterica* and *S. aureus*. Together, these findings demonstrate that each of the four treponemal peptides identified in our AMP discovery bioinformatics pipeline are capable of exhibiting both bacteriostatic and bactericidal activity.

**TABLE 4:** Antimicrobial susceptibility testing of *T. pallidum* candidate AMPCCRs using broth microdilution.

Peptide	*E. coli* ATCC 9723H		*P. aeruginosa* ATCC 10148		*S. enterica* SL1344		*S. aureus* ATCC 6538P		*S. pyogenes* Unknown strain; Clinical Isolate		*M. smegmatis* MC^2^155	
	MIC	MBC	MIC	MBC	MIC	MBC	MIC	MBC	MIC	MBC	MIC	MBC
Tp0451a_N	–	–	–	–	–	–	–	–	–	–	34.7–69.5	69.5
Tp0451a_C	–	–	–	–	–	–	–	–	5.4–10.8	5.4–43.3	1.4–2.7	1.4–5.4
Tp0749_N	–	–	–	–	–	–	–	–	–	–	0.3–1.3	1.3
Tp0749_C	12.0	48.2–96.4	24.1–48.2	96.4	–	–	–	–	–	–	1.5–3.0	1.5–3.0
Tp0451a_C_C85S	10.9–21.8	10.9–43.6	–	–	–	–	–	–	5.4–10.9	10.9–43.6	5.4	10.9
Tp0749_C_C61S	24.2–48.5	–	97.0	–	–	–	–	–	–	–	12.1–24.2	24.2–48.5
Tp0751_p5 (–ve)	–	–	–	–	–	–	–	–	–	–	–	–
sLL-37 (–ve)	–	–	≥57	–	–	–	–	–	–	–	14.2–28.5	≥57
LL-37 (+ve)	1.8–7.1	1.8–7.1	3.6–14.2	3.6–14.2	1.8–28.5	1.8–28.5	7.1–28.5	7.1–57.0	28.5–57.0	28.5–57.0	0.45–1.8	0.45–1.8
RaCa-2 (+ve)	3.1–12.4	6.2–24.7	49.4–98.9	≥98.9	6.2–49.4	12.4–98.9	3.1–6.2	3.1–12.4	6.2–49.4	6.2–49.4	1.5–3.1	1.5–6.2

*All peptides were tested between three and nine times in independent experiments against a panel of six clinically and biologically relevant Gram-negative, Gram-positive, and Mycobacterium species. MIC and MBC ranges (μM) are shown for each peptide. Strains are indicated for each bacterium with the exception of S. pyogenes (clinical isolate, unknown strain). -ve, negative control peptide; +ve, positive control peptide; –, no AMP activity.*

Based on our six-member panel of bacteria, we found Tp0749_C to be the most broad-spectrum AMP of the four treponemal peptides. In addition to its anti-mycobacterial properties, it also showed moderate bacteriostatic activity towards *E. coli*, moderate-low bacteriostatic activity against *P. aeruginosa*, and low bactericidal activity against both *E. coli* and *P. aeruginosa* (Table 4). The positive control peptide, LL-37, was more active against *E. coli* and *P. aeruginosa* than Tp0749_C. The positive control peptide, RaCa-2, was more active against *E. coli* than Tp0749_C but exhibited lower anti-pseudomonal activity. The other three treponemal peptides showed no activity against these two Gram-negative bacteria. The negative control peptide, Tp0751_p5, a 24-mer peptide from the *T. pallidum* adhesin Tp0751 with similar physicochemical properties to AMPs, was inactive against all six bacteria in all experiments. Surprisingly, the negative control peptide, sLL-37, a scrambled version of LL-37, exhibited a low degree of antimicrobial activity against *M. smegmatis* and minimal bacteriostatic activity against *P. aeruginosa* (Table 4). Since sLL-37 retains many of the same physicochemical properties, including overall charge and amino acid composition, as LL-37, but is predicted to lack the helical content found in native LL-37, this may explain the low-level activity observed with the scrambled version of this peptide. Indeed, a similar low level of antimicrobial activity for sLL-37 has been demonstrated previously in an independent study (Gordon et al., 2005). Together, the Tp0451a and Tp0749 results suggest that *T. pallidum* is capable of producing AMPs that target Gram-negative bacteria, Gram-positive bacteria, and mycobacteria, and established proof-of-concept for our AMP discovery bioinformatics pipeline.

To evaluate if cysteine residues are important for *T. pallidum* AMP function, cysteine-to-serine substituted versions of Tp0451a_C (Tp0451a_C_C85S) and Tp0749_C (Tp0749_C_C61S) were synthesized and tested for antimicrobial activity using our panel of six bacteria and broth microdilution assays. These peptides were chosen as they have broad-spectrum AMP activity, and only contain one cysteine residue each. As shown in Table 4, compared to the unmodified, cysteine-containing AMPCCR, Tp0451a_C, the antimicrobial activity of Tp0451a_C85S against *M. smegmatis* was reduced two- to eight-fold, whereas the cysteine substitution had no effect on anti-streptococcal activity. Interestingly, the cysteine substituted version of Tp0451a_C exhibited moderate antimicrobial activity against *E. coli*, unlike the unmodified version. Compared to the unmodified, cysteine-containing AMPCCR, Tp0749_C, the bacteriostatic activity of Tp0749_C_C61S against *E. coli* and *P. aeruginosa* was reduced two- to four-fold, and reduced eight-fold against *M. smegmatis* (Table 4). Furthermore, the low bactericidal activity of Tp0749_C against *E. coli* and *P. aeruginosa* was abolished in Tp0749_C_C61S and the strong bactericidal activity against *M. smegmatis* was reduced 16-fold. Consistent with the high abundance of cysteines in the top-ranking predicted AMPs, these findings show that the cysteine residues are important for the AMP activity.

To investigate the potential antimicrobial activity of the *T. pallidum* AMPCCRs against the frequently co-infecting sexually transmitted pathogen, *N. gonorrhoeae*, an antimicrobial susceptibility assay based on agar dilution was developed and performed to test for bacteriostatic and bactericidal activities. Although growth was visibly inhibited by Tp0451a_C, none of the four treponemal AMPCCRs completely inhibited *Neisseria* growth on the agar plates at any of the peptide concentrations. The visible inhibition of growth by Tp0451a_C prompted us to test whether any of the treponemal peptides were bactericidal against *N. gonorrhoeae* by comparing TVCs of the 3 h-incubated peptide/bacteria mixtures with TVCs from the corresponding positive control growth wells to give the percentage of bacteria killed by the peptides. In agreement with our modified agar dilution results, we found Tp0451a_C to be the only treponemal peptide capable of exhibiting bactericidal activity against *N. gonorrhoeae*, with *Neisseria*-killing activity consistently observed at peptide concentrations of 64 μg/mL and higher (Table 5). The loss of anti-*Neisseria* activity in the cysteine substituted version of Tp0451a_C (Tp0451a_C_C85S) further suggested the importance of cysteine residues in treponemal AMP function. These findings suggest that *T. pallidum* may express proteins that are capable of killing *Neisseria* during co-infections involving these two sexually transmitted pathogens.

**TABLE 5:** Antimicrobial susceptibility testing of *T. pallidum* candidate AMPCCRs against *N. gonorrhoeae* using a modified agar dilution method.

Peptide	256 μg/mL	128 μg/mL	64 μg/mL	32 μg/mL

	***N. gonorrhoeae* killing (%)**			
Tp0451a_N	0	0	nt	nt
Tp0451a_C	97.6–100	94.4–100	90.5–100	0–93.0
Tp0749_N	0	0	nt	nt
Tp0749_C	0	0	nt	nt
Tp0451a_C_C85S	0	0	0	0
Tp0749_C_C61S	0	0	nt	nt
Tp0751_p5 (-ve)	0	0	0	0
sLL-37 (-ve)	0	0	0	0
LL-37 (+ve)	100	100	100	100
RaCa-2 (+ve)	100	100	100	100

*The bactericidal activity of treponemal peptides was analyzed by comparing TVCs following a 3-h incubation period in the presence of N. gonorrhoeae with the TVCs from the positive growth control samples (N. gonorrhoeae, no peptides present). All peptides were tested in three to six independent experiments against N. gonorrhoeae (ATCC 700825, streptomycin resistant). The percentage killing of N. gonorrhoeae from all experiments is shown for each of the peptides. nt, not tested.*

### AMPCCR Susceptibility Testing of *T. pallidum*

*Treponema pallidum* exhibits vigorous motility which can be used as an indication of bacterial viability. To assess the activity of the treponemal peptides against *T. pallidum*, *in vitro*-cultured *T. pallidum* was incubated with each peptide at three different concentrations. Treponeme viability was then determined by counting motile treponemes using darkfield microscopy. As shown in Supplementary Figure 5, only Tp451a_C showed antimicrobial activity against *T. pallidum* at 64, 16, and 4 μg/ml peptide concentrations, when compared to the negative control peptide, Tp0751_p5. The positive control peptide, LL-37, and Tp0749_N also showed a low level of inhibitory activity against *T. pallidum* at 64 μg/ml. These findings demonstrate that, similar to other bacteria (Beis and Rebuffat, 2019; Smits et al., 2020), *T. pallidum* is susceptible to the antimicrobial activity of some of the AMPs it produces, at least when added exogenously.

### Immunomodulatory Capabilities of *T. pallidum* AMPCCRs

Based upon the well-established propensity for AMPs to suppress or induce cytokine production and the persistent nature of *T. pallidum* infection, we assessed potential immunomodulatory functions of the *T. pallidum* AMPCCRs through examining their capacity to influence cytokine production (IL-1β, IL-6, IL-8, IL-10, MCP-1, and TNF) from a human monocyte/macrophage cell line. Initially, we quantified cytokine production from monocytes (THP-1 cells) stimulated with the treponemal peptides under non-inflammatory conditions. Stimulation with treponemal peptides resulted in the production of several cytokines from monocytes, but the levels of cytokine were low (Supplementary Figure 6). No statistically significant differences in cytokine production between monocytes stimulated with Tp0751_p5 (peptide with no antimicrobial activity in the present study), and the treponemal AMPCCR, Tp0451a_C, was detected. Exposure of each of the peptides to macrophages (differentiated THP-1 cells) failed to induce a significant difference in cytokine expression when compared to the unstimulated control (Supplementary Figure 7). In summary, no treponemal AMPCCR-specific effect on cytokine secretion by undifferentiated monocytes or macrophages was observed in these non-inflammatory immunomodulation assays.

**FIGURE 7:**
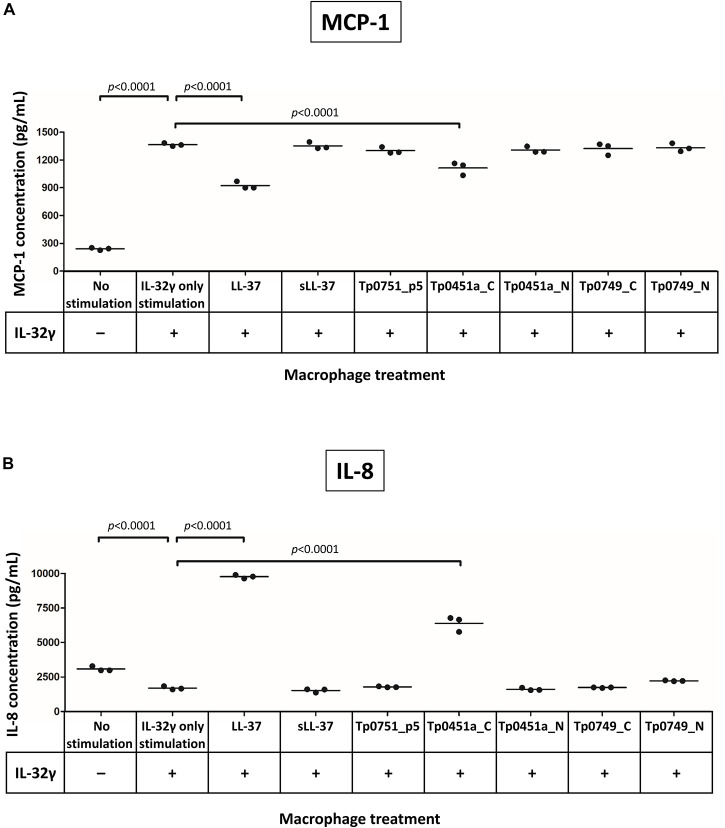
Analysis of the immunomodulatory capacities of the *T. pallidum* AMPCCRs. Human THP-1 cells were differentiated to macrophages and then stimulated with or without IL-32γ. Cells were then immediately exposed to either LL-37, sLL-37, Tp0751_p5, Tp0451a_C, Tp0451a_N, Tp0749_C, or Tp0749_N and analyzed for expression of **(A)** MCP-1 and **(B)** IL-8. Each data point is representative of cells from one well of a 12-well plate. Data shown is representative of three independent experiments. A Dunnett’s multiple comparisons test was used for normally distributed data and a Dunn’s multiple comparisons test was used for data that was not normally distributed. For statistical analyses, mean values from each peptide were compared to the mean of the unstimulated control (IL-32γ only stimulation); *p*-values (Dunnett’s multiple comparisons test) indicating statistically significant differences observed in three independent experiments are indicated.

Previously, it has been shown that LL-37 modulates macrophage cytokine production under conditions where macrophages have been highly activated by exposure to the pro-inflammatory cytokine IL-32γ (Choi et al., 2014). IL-32γ also plays key roles in pathogen defense and persistence of chronic infection (Bai et al., 2010; Dos Santos et al., 2017; Li et al., 2018). To assess whether the *T. pallidum* AMPCCRs identified in the current study also modulate cytokine expression levels under cytokine-induced pro-inflammatory conditions, macrophages were activated with IL-32γ and immediately additionally stimulated with one of our control peptides (LL-37, sLL-37, and Tp0751_p5), or one of the four treponemal AMPCCRs (Tp0451a_N, Tp0451a_C, Tp0749_N, and Tp0749_C), or not exposed to a peptide (IL-32γ only) or IL-32γ (no stimulation). IL-32γ elicited robust production of the chemokine MCP-1 from macrophages compared to non-IL-32γ stimulated cells, demonstrating that the cells were successfully activated, and notably, both LL-37 and the treponemal AMPCCR Tp0451a_C were found to be the only peptides that significantly downregulated (*p* < 0.0001 for both peptides) IL-32γ-induced expression of MCP-1, in three independent experiments (Figure 7A). Additionally, LL-37 and Tp0451a_C were able to modulate macrophage-production of the chemokine IL-8 following IL-32γ stimulation: IL-32γ stimulation resulted in lower levels of IL-8 release compared to unstimulated cells, and co-stimulation of macrophages with IL-32γ and LL-37 or Tp0451a_C caused significantly higher levels of IL-8 release compared to from macrophages stimulated with IL-32γ alone (*p* < 0.0001 for both peptides in three independent experiments (Figure 7B). Co-stimulation of macrophages with each of the peptides failed to significantly affect the expression levels of IL-1β, IL-6, TNF, or IL-10 (Supplementary Figure 8). In addition to the antimicrobial activities described above, these findings demonstrate that the *T. pallidum* AMPCCR Tp0451a_C is also capable of immunomodulatory activities in certain inflammatory contexts.

## Discussion

In this study, we investigated the potential for *T. pallidum* to express AMPs as a previously unrecognized strategy to defend against competing bacteria and the host immune response. To conduct these studies, we developed a bioinformatics pipeline for the identification of candidate *T. pallidum* AMPs. By using AMPCCR mapping, followed by a combination of structure, modeling, homology, and cell penetration prediction analyses, potential AMPCCRs were identified in two *T. pallidum* proteins, Tp0451a and Tp0749. Together, these findings enabled the design and synthesis of four putative AMPCCRs to evaluate these *T. pallidum* proteins for antibacterial and immunomodulatory functions.

Using antimicrobial susceptibility assays, we demonstrated bacteriostatic and bactericidal activity for the four predicted *T. pallidum* AMPCCRs. All four peptides were active against the model *Mycobacterium* sp., *M. smegmatis*, three of which (Tp0451a_C, Tp0749_N, and Tp0749_C) exhibited robust anti-mycobacterial activity that was similar or more potent than the activity of many antimycobacterial AMPs from other organisms (Abedinzadeh et al., 2015; Gupta et al., 2015; Helbing et al., 2019). *Mycobacterium smegmatis* is generally considered as nonpathogenic, however, it has been shown on rare occasions to cause disease in humans (Wallace et al., 1988; Newton et al., 1993; Pennekamp et al., 1997; Hong et al., 2003; Ergan et al., 2004). Interestingly, *M. smegmatis* was originally isolated from syphilitic chancres and gummas (Bloom, 1885) and is known to be present in normal genital (smegma) secretions. *Mycobacterium smegmatis* is neither a true Gram-negative nor Gram-positive due to its unusual cell envelope ultrastructure and composition, which is similar in all known mycobacteria species, including the important human pathogen *Mycobacterium tuberculosis* (Brennan and Nikaido, 1995; Cook et al., 2009). Although few cases have been documented, *T. pallidum*/*M. tuberculosis* coinfections in HIV patients have been reported (Latif et al., 2020). Given the cell envelope conservation observed within mycobacteria, the *T. pallidum* proteins demonstrated in this study to possess AMP activity may also be capable of targeting *M. tuberculosis* during situations of co-infection.

One of the treponemal AMPCCRs (Tp0451a_C) exhibited bacteriostatic and bactericidal activity against a clinical isolate of the Gram-positive extracellular bacterial pathogen, *S. pyogenes*. At the time of writing, searches of the AMP database, APD3 (see footnote 26) (Wang et al., 2016), identified only 71 known AMPs (out of a total of 3324 known AMPs) with antimicrobial activity against *S. pyogenes*, including only 12 from other bacteria, most of which are non-pathogenic members of the microbiota. In line with these results, few reports have been published describing this rare antimicrobial activity, making Tp0451a_C a new addition to this select group of *S. pyogenes*-targeting AMPs. Furthermore, the anti-*S. pyogenes* activity of Tp0451a_C was found to be generally comparable (Cogen et al., 2010; Uhlmann et al., 2016; Ma et al., 2020) or more potent (Sornwatana et al., 2018; Li et al., 2022) than known AMPs from other organisms. The relatively small number of AMPs that show activity against *S. pyogenes* is attributed to the expression of several proteins involved in AMP resistance, including the streptococcal cysteine protease SpeB (Schmidtchen et al., 2002) which is involved in the proteolytic degradation and inactivation of AMPs, the M1 protein, streptokinase, and the streptococcal inhibitor of complement, the latter three of which are involved in resistance against defensins and/or LL-37 (Frick et al., 2003; Lauth et al., 2009; Hollands et al., 2012). Our findings show that the AMP-activity identified in Tp0451a_C is capable of circumventing the resistance mechanisms developed by pathogenic *S. pyogenes*. Importantly, *S. pyogenes* colonizes and infects several host sites that *T. pallidum* also encounters, particularly during *T. pallidum* transmission. These shared host sites include the skin and the mucosal membranes of the oropharynx, rectum, and genitals (Stamm et al., 1978; Mead and Winn, 2000; Lafond and Lukehart, 2006; Sobel et al., 2007; Minami et al., 2010; Verstraelen et al., 2011; Nelson et al., 2016; Norimatsu and Ohno, 2020). Taken together, these findings suggest that *Streptococcus*-killing AMPs may comprise a defense strategy that facilitates survival during infection by protecting *T. pallidum* against this clinically relevant, competing microbe in key host infection sites such as the genital mucosa, via direct inhibition and killing.

Tp0451a_C was also demonstrated to be the only treponemal peptide capable of exhibiting bactericidal activity against the frequently co-infecting sexually transmitted pathogen *N. gonorrhoeae* (Bala et al., 2011). This finding is consistent with the concept that *T. pallidum* and *N. gonorrhoeae* localize to the same environmental niches within the urogenital region, particularly during transmission and primary stage syphilis when treponemes are localized within the chancre at the initial site of infection. In light of the increasing global public health threat posed by *N. gonorrhoeae* due to the rapid emergence of multiple drug resistant strains and with an estimated 78 million new cases per year (Alirol et al., 2017), our finding of a *Neisseria*-active AMP is relevant to the goal of developing novel therapeutics against this pathogen.

Most AMPs from bacteria exhibit narrow antimicrobial spectra; they are produced in order to defend themselves and their environmental niche from a few genus/species-specific competing species that are often also closely related to the AMP-producing bacterium (Cleveland et al., 2001; Cotter et al., 2005; De Vuyst and Leroy, 2007). This is most likely because closely related bacterial species often reside within the same environmental niches. However, identification of broad spectrum AMPs has become increasingly common, particularly amongst non-pathogenic Gram-positive bacteria (Kemperman et al., 2003a,b; Zendo, 2013; Todorov et al., 2019). Here we found Tp0749_C and Tp0451a_C to exhibit the most broad-spectrum activity of the four treponemal peptides tested. Interestingly, Tp0749_C exhibited bacteriostatic and bactericidal activity against a non-pathogenic strain of the Gram-negative bacterium, *E. coli*. Non-pathogenic *E. coli* strains are normal, prevalent residents of the human lower gastrointestinal tract, including the rectum (Zhang et al., 2002; Tenaillon et al., 2010). The fact that syphilis chancres commonly occur in the rectum and anal canal of men-who-have-sex-with-men infected with *T. pallidum* (Lafond and Lukehart, 2006), suggests (i) the likelihood of a close association between non-pathogenic *E. coli* and *T. pallidum* during primary stage syphilis in these individuals and (ii) the possibility that *T. pallidum* AMPs are expressed that target non-pathogenic *E. coli* in this host infection site. It is also likely that *T. pallidum* encounters pathogenic / uropathogenic strains of *E. coli* during infection. Although these strains were not tested in our antimicrobial susceptibility assays, previous studies have shown that AMPs from other organisms exhibit similar levels of antimicrobial activity against both non-pathogenic and pathogenic / uropathogenic *E. coli* strains (Fedders et al., 2010; Aghazadeh et al., 2019; Mardirossian et al., 2019; Moazzezy et al., 2020; Li et al., 2022; Lin et al., 2022). Tp0749_C also exhibited antimicrobial activity against the Gram-negative bacterium, *P. aeruginosa*. *P. aeruginosa* is an opportunistic pathogen that is the causative agent of both severe acute and chronic infections in immunocompromised individuals, and is one of the major etiological agents of urinary tract infections (Ronald, 2002; Shigemura et al., 2006). Given that *T. pallidum* / HIV co-infections are common (Shockman et al., 2014; Burchell et al., 2015), and the potential for co-localization of *T. pallidum* and *P. aeruginosa* to the same environmental niches within the urogenital region in immunocompromised patients, these findings support the concept that *T. pallidum* may produce anti-pseudomonal AMPs as a defense mechanism during infection. Together, T0451a and Tp0749 were capable of inhibition and/or killing of five of the seven diverse bacterial species tested, including three Gram-negative bacteria, a Gram-positive bacterium, and a mycobacterium. This finding is consistent with the ability of *T. pallidum* to invade and disseminate to any host organ or tissue during the different stages of syphilis and the variable, and often complex, polymicrobial environments that exist within different sites of infection.

As part of our physicochemical analyses of *T. pallidum* miniproteins, we determined that the rare amino acid cysteine appears frequently in high-ranking predicted AMPs. Substitution of the single cysteine residue in Tp0451a_C with the structurally similar residue serine reduced its antimicrobial activity against *N. gonorrhoeae* and *M. smegmatis*, but resulted in a gain of antimicrobial activity against *E. coli*. Substitution of the single cysteine residue in Tp0749_C with serine greatly reduced the antimicrobial activity against *E. coli* and *P. aeruginosa*, and even more so against *M. smegmatis*. This unusual physicochemical property is shared with specific classes of AMPs, in particular certain eukaryotic AMPs, including defensins which typically contain six to twelve cysteines (Shafee et al., 2016), protegrins, which are active against several sexually transmitted pathogens and often contain four cysteines (Tamamura et al., 1995; Fahrner et al., 1996; Qu et al., 1996; Yasin et al., 1996), and brevinins which usually contain two cysteine residues (Simmaco et al., 1994). More recently, increasing numbers of cysteine-rich and defensin-like AMPs are being discovered in bacteria (Baindara et al., 2017; Sugrue et al., 2020). The even number of cysteine residues in these AMPs allows for the formation of intra-molecular disulfide bonds, which are important for AMP stability (Dias Rde and Franco, 2015). Literature and PDB searches indicate that most cysteine-containing AMP structures that have been solved are monomers that contain even numbers of cysteines / intra-molecular disulfide bonds. However, some AMPs with an odd number of cysteines form inter-molecular disulfide bonds resulting in dimers that can increase activity and stability compared to monomeric forms (Campopiano et al., 2004; Min et al., 2017). The AMPCCRs, Tp0451a_C and Tp0749_C, should only be capable of forming inter-molecular disulfide bonds / multimers, as they only contain one cysteine each. However, full-length Tp0451a_C contains seven cysteine resides and Tp0749 contains two cysteine residues, suggesting the additional possibility of intra-molecular disulfide bond formation. Similar to the aforementioned cysteine-rich AMPs, our findings are in agreement with the hypothesis that cysteines are important for the demonstrated treponemal AMP activity, possibly via the formation of inter- and/or intra-molecular disulfide bonds and miniprotein multimerization.

Due to the antimicrobial findings described herein, the fact that many AMPs have the ability to modulate immune functions (Lai and Gallo, 2009; Kindrachuk et al., 2013; Dicks et al., 2018; Malaczewska et al., 2019), and the stealth nature of *T. pallidum*, we investigated the immunomodulatory capabilities of the four treponemal peptides under both non-inflammatory and cytokine-induced inflammatory conditions, through assaying cytokine production from a human monocyte/macrophage cell line. Given that IL-32γ plays key roles in pathogen defense and persistence of chronic infection (Bai et al., 2010; Dos Santos et al., 2017; Li et al., 2018), and that AMP-mediated immunomodulatory activities have been shown to modulate IL-32γ-induced cytokine production (Choi et al., 2014), IL-32γ was selected as a biologically relevant co-stimulatory agent for our assays to mimic the pro-inflammatory environment present during *T. pallidum* infection (Lafond and Lukehart, 2006). Interleukin-32 is a recently discovered pro-inflammatory cytokine that functions in the persistence of inflammation via induction of other pro-inflammatory cytokines and in the control of infectious and chronic diseases (Khawar et al., 2016; Ribeiro-Dias et al., 2017). It has been found to be elevated in human infections, where it can have a protective (*Mycobacterium tuberculosis*, HIV) or detrimental (*Helicobacter pylori*) effect on the host (Rasool et al., 2008; Bai et al., 2010; Peng et al., 2014). Although the up-regulation and involvement of IL-32 isoforms in *T. pallidum* infection has yet to be demonstrated, the inflammation associated with *T. pallidum* infection is consistent with this possibility. Furthermore, IL-32 is upregulated in HIV infection (Rasool et al., 2008), a pathogen that is frequently involved in co-infections with *T. pallidum*.

Under IL-32γ-induced pro-inflammatory conditions in macrophages, we found that co-stimulation with Tp0451a_C resulted in decreased production of the pro-inflammatory chemokine MCP-1 and increased production of the pro-inflammatory chemokine, IL-8, compared to macrophages not exposed to peptides. MCP-1 is produced by a variety of cell types, several of which are relevant to *T. pallidum* infection, including monocytes, macrophages, epithelial, and endothelial cells (Yoshimura et al., 1989a,b; Cushing et al., 1990; Standiford et al., 1991). Monocyte chemoattractant protein-1 is the main chemoattractant for monocytes and macrophages to sites of inflammation where recruited monocytes undergo transformation into macrophages (Deshmane et al., 2009). During infection, clearance of *T. pallidum* is dependent on macrophages via antibody-mediated opsonophagocytosis and subsequent killing by macrophages (Lukehart and Miller, 1978; Baker-Zander and Lukehart, 1992; Baker-Zander et al., 1993). However, in the absence of antibiotic intervention, treponemes are never fully cleared from the host. The ability of Tp0451a to down-regulate expression of pro-inflammatory cytokines, including MCP-1, may contribute to treponemal survival via local suppression of the inflammatory response and dampening of macrophage recruitment.

In the present study, we also found Tp0451a_C capable of significantly increasing production of IL-8 in co-stimulated macrophages. This pro-inflammatory cytokine is a potent neutrophil chemoattractant that can induce neutrophil morphology changes and degranulation, and neutrophil migration to sites of infection and inflammation (Baggiolini and Clark-Lewis, 1992; Kolaczkowska and Kubes, 2013; Arango Duque and Descoteaux, 2014). Similar to the findings described herein, IL-8 has been shown to be up-regulated in macrophages stimulated with *T. pallidum* peptides derived from the major immunogenic lipoprotein, TpN47 (Sellati et al., 1998). If IL-8 up-regulation, by Tp0451a_C or other treponemal proteins, is recapitulated at the level of viable whole *T. pallidum*, the following consequences may be envisioned that could promote *T. pallidum* pathogenesis. Since neutrophils are largely ineffective against *T. pallidum* during early infection (Lafond and Lukehart, 2006), IL-8 production may function as an early infection immune diversion mechanism. Alternatively, an increase in IL-8 expression may redirect the immune response towards competing neutrophil-sensitive microorganisms in polymicrobial host sites, such as uropathogenic *E. coli* (Svanborg-Eden et al., 1987). As IL-8 is also a pro-angiogenic chemokine (Li et al., 2003), up-regulation may contribute to the vascular inflammation and increased angiogenesis observed during secondary syphilis (French, 2007; Gao et al., 2019) and may promote treponemal accessibility to the host’s circulatory system via proliferation of endothelial cells and formation of new blood vessels, similar to the mechanism proposed for the IL-8-inducing *T. pallidum* protein TpF1 (Pozzobon et al., 2016).

Here we employed a combination of bioinformatics, antimicrobial susceptibility testing, and immunomodulation assays that allowed for the experimental identification of the first proteins with AMP activity from a spirochete bacterium. However, there are limitations with our approach. First, due to difficulties with expression, full-length versions of the treponemal proteins were not used in antimicrobial susceptibility or immunomodulation experiments. Confirmation of AMP activity with the full-length proteins would be optimal, and may uncover an enhanced AMP activity due to the additive effects of the AMPCCRs. Second, in our antimicrobial susceptibility assays, we only tested for antibacterial activity and thus may have missed antifungal, antiviral, and/or antiparasitic activities. Third, although most AMPs are smaller than 150 amino acids, our bioinformatics pipeline omitted larger proteins from our analyses thereby excluding potential colicin-like bacteriocins. Fourth, the expression status of approximately three-quarters of all *T. pallidum* miniproteins of unknown function remain to be determined, due in part to the experimental difficulty with identifying small, positively-charged proteins via mass spectrometry-based proteomic analyses. Furthermore, the identification of potentially secreted *T. pallidum* proteins from *in vivo* and *in vitro* cultures by mass spectrometry and other methodologies is not possible due to the overwhelming abundance of contaminating rabbit/host proteins and the complexity of the culture medium, respectively. Fifth, similar to other AMP studies, the *in vitro* nature of the antimicrobial susceptibility assays, which were based on established, standardized protocols (Wiegand et al., 2008), are not representative of the complex host environments encountered during infection. Therefore, it remains to be determined if the concentrations of *T. pallidum* peptides that resulted in bacteriostatic and bactericidal activities are representative of the concentrations that are expressed and secreted into diverse host sites during infection.

Finally, and most significantly, similar to other recently discovered AMPs, our functional characterization of the treponemal proteins with AMP activity has yet to determine if/how they are processed/activated, and the mechanisms involved in self-immunity and export. Most bacterial AMPs that have been functionally characterized are synthesized as inactive protein precursors comprised of a N-terminal leader peptide/signal peptide, that is required for export, and a C-terminal proprotein that contains the critical core regions (Torrent et al., 2009, 2012; Chang et al., 2015; Perez et al., 2018). Following specific protease-mediated cleavage of the leader / signal peptide, the mature, functionally-active AMP is exported from the AMP-producing bacteria to the external environment via ATP-binding cassette (ABC) transporters (Beis and Rebuffat, 2019). In the present study, we showed that several *T. pallidum* candidate AMPs contain N-terminal amino acid sequences with similarity to the Gram-negative Sec-independent double-glycine/glycine-alanine (GG/GA) leader peptides, suggesting that a subset of *T. pallidum* AMPs may also use this recognition signal to direct secretion and allow for activation of precursor AMP forms. In addition, proteome functional annotation analyses and Phyre2 structure modeling of open reading frames located close to several potential AMPs identified (1) putative homologs and structural orthologs of proteases with potential roles in AMP leader peptide cleavage and activation and (2) ABC transporters that may mediate export of the mature active treponemal AMPs to the external host environment during infection. Notably, an emerging class of bacterial AMPs with varying characteristics, referred to as leaderless AMPs, do not undergo post-translational processing or modification, are produced without an export-mediating N-terminal leader/signal peptide, and are fully active immediately following expression (Perez et al., 2018). Leaderless AMPs are the most poorly understood group of bacterial AMPs, and the molecular mechanisms underlying self-immunity, secretion, and export remain largely unknown (Perez et al., 2018). Although no common secretion or export mechanism has been discovered for leaderless AMPs, an ABC transporter protein (LmrB) has been shown to be involved in the immunity and export of a leaderless AMP from *Lactococcus lactis*, LsbB (Gajic et al., 2003). Additionally, an ABC transporter protein (DDHIJ) from *Enterococcus faecalis* has been implicated in the active export of the leaderless AMP, Enterocin DD14 (Ladjouzi et al., 2020). It is possible that Tp0451a and Tp0749 belong to, or are related to, the leaderless group of bacterial AMPs. In this situation, the two *T. pallidum* AMPs would be expressed without processing/proteolytic cleavage, followed by export via one or more of the many ABC transporter proteins located within the *T. pallidum* proteome (Fraser et al., 1998). This mechanism would result in the N- and C-terminal AMPCCRs identified in the current study to remain fully intact and associated with each other, potentially allowing for enhanced, broad spectrum AMP activity due to the additive effects of the AMPCCRs. This is consistent with the knowledge that AMP activity is often localized to more than one discrete peptide region, or domain (AMPCCRs), within the full-length protein (Todorov, 2009; Torrent et al., 2012; Snyder and Worobo, 2014).

In the present study, we showed that the viability of *T. pallidum* was reduced following incubation with Tp0451a_C. This result was not surprising, given that AMPs from other bacteria are highly toxic to the AMP-producing strain, necessitating the expression of self-immunity proteins and dedicated ABC transporters that protect the AMP-producing bacteria during expression within the cell through extrusion outside and away from the cell (Smits et al., 2020). The unique composition of the *T. pallidum* cell envelope may confer a degree of protection against AMPs, as suggested in a previous study that investigated the activity of the full-length mammalian AMP LL-37 against *T. pallidum* (Cox et al., 2003). Specifically, the *T. pallidum* outer membrane has low anionic phospholipid content compared to other Gram-negative bacteria, contains few outer membrane proteins including porins and negatively-charged proteins, lacks LPS, and contains cholesterol. Thus, *T. pallidum* possesses a cell envelope that is reminiscent of the host cell membrane with regards to a neutral surface charge and the presence of the AMP inhibitory molecule, cholesterol (Feigin et al., 1995; Matsuzaki et al., 1995; Glukhov et al., 2005). Together, these may help prevent the electrostatic binding of cationic AMPs to the *T. pallidum* surface, which is consistent with our findings in the current study that showed only minor or no decrease in *T. pallidum* viability following incubation with all but one of the AMPs that were tested.

In conclusion, this study has established proof-of-concept for our AMP discovery bioinformatics pipeline via the experimental identification of proteins with AMP activity in *T. pallidum*. The ability of *T. pallidum* to produce proteins with dual antimicrobial and immunomodulatory activities may contribute to treponeme survival by eliminating competing microbes via direct inhibition and killing effects, and by modulating the host immune response to promote both indirect inhibition and killing of competing bacterial species and immune evasion. This research has the potential to enhance our understanding of the unique pathogenesis of *T. pallidum* and reveal novel defense and survival mechanisms with broad applicability to bacterial pathogens, including other pathogenic spirochetes.

## Data Availability Statement

The original contributions presented in the study are included in the article/Supplementary Material, further inquiries can be directed to the corresponding author.

## Ethics Statement

The animal study was reviewed and approved by the local institutional review board at the University of Victoria, and conducted in strict accordance with standard accepted principles as set forth by the Canadian Council on Animal Care, National Institutes of Health and the United States Department of Agriculture in a facility accredited by the Canadian Council on Animal Care and the American Association for the Accreditation of Laboratory Animal Care.

## Author Contributions

SH, CC, LR, KC, ES, RR, and MB contributed to the experimental design. SH, KC, ES, AG, SM, AH, and RR conducted the experiments. SH, KC, RR, ES, LR, MB, and CC were involved in the analysis and interpretation of the data. CC and LR acquired financial support for the project. SH wrote the first draft of the manuscript with contributions from KC. ES, RR, AG, SM, AH, CC, LR, and MB reviewed the manuscript before submission for accuracy and intellectual content. All authors contributed to the article and approved the submitted version.

## Conflict of Interest

The authors declare that the research was conducted in the absence of any commercial or financial relationships that could be construed as a potential conflict of interest.

## Publisher’s Note

All claims expressed in this article are solely those of the authors and do not necessarily represent those of their affiliated organizations, or those of the publisher, the editors and the reviewers. Any product that may be evaluated in this article, or claim that may be made by its manufacturer, is not guaranteed or endorsed by the publisher.

## Footnotes

1
https://www.ncbi.nlm.nih.gov/genome/?term=treponema+pallidum

2
https://www.dveltri.com/ascan/

3
http://cabgrid.res.in:8080/amppred/

4
http://www.camp.bicnirrh.res.in/predict

5
https://services.healthtech.dtu.dk/service.php?SignalP-5.0

6
https://services.healthtech.dtu.dk/service.php?LipoP-1.0

7
https://www.ebi.ac.uk/Tools/msa/clustalo/

8
https://weblogo.berkeley.edu/logo.cgi

9
https://aps.unmc.edu/prediction

10
http://www.sbg.bio.ic.ac.uk/~phyre2/html/page.cgi?id=index

11
http://tcoffee.crg.cat/apps/ampa/do

12
http://www.camp.bicnirrh.res.in/predict_c/

13
https://webs.iiitd.edu.in/raghava/antibp/submit.html

14
https://webs.iiitd.edu.in/raghava/antibp2/submit.html

15
http://www.compbio.dundee.ac.uk/jpred/

16
http://bioinf.cs.ucl.ac.uk/psipred/

17
http://heliquest.ipmc.cnrs.fr/

18
https://mobyle.rpbs.univ-paris-diderot.fr/cgi-bin/portal.py#forms::PEP-FOLD

19
https://swissmodel.expasy.org/

20
https://salilab.org/modeller/

21
https://www.molsoft.com/index.html

22
http://prodata.swmed.edu/promals3d/promals3d.php

23
https://blast.ncbi.nlm.nih.gov/Blast.cgi

24
https://webs.iiitd.edu.in/raghava/cellppd/submit_prot.php

25
https://aps.unmc.edu/
