# Supplementary material for: Identification and Functional Characterization of Peptides With Antimicrobial Activity From the Syphilis Spirochete, Treponema pallidum
Source: Front Microbiol. 2022 May 3;13:888525. doi: 10.3389/fmicb.2022.888525 (PMC9200625; doi:10.3389/fmicb.2022.888525)
Supplement: Supplementary file 7 [file Data_Sheet_1.PDF]

Supplementary Figure S1

> Tp0360 TPANIC_RS05425 WP_014342791.1 (RANK 33/68)
4 / 23 amino acid peptide
MAGASKNSRTAAATQRFNCPCGGEVVLRSIVDNGKVKNIAECPKCRRVERRPRDFN

> Tp0451 TPANIC_RS05285 WP_010881899.1 (RANK 68/68)
14 amino acid peptide
MLSMQLVTVTVQGAMVADIAVAVRTAPHPFLYASVLQDSPQARKPVRETRYQKMSAFLCT

> Tp0749 TPANIC_RS05345 WP_010882194.1 (RANK 24/68)
10 amino acid peptide
MQDKALFSGALDTPFMQVITWARLYHKNQKRYEKIKKSFTFHETCLQSTKGIVAERILKPCVRRKVNGKF
RST

> Tp0084 TPANIC_RS00410 WP_010881533.1 (RANK 63/68)
9 amino acid peptide
MVVNAVVGADEASARLREYCSGLPDVEKKIAESTSPEGAKLVSDFGIGSVPMVVILDEDSSELFRTADIG
ELEKFFS

> Tp0869 TPANIC_RS04290 WP_010882312.1 (RANK 19/68)
17 amino acid peptide
MRSCPKRARGVHWATGAALWCPSRMIFEKISPLQAFVWAVLRLFLKSFRTVFRGAVRAGCGVLACVRAYG
FPPYGSKE

> Tp0352 TPANIC_RS01725 WP_010881800.1 (RANK 32/68)
7 amino acid peptide
MREKEGGVVNDDFHYEVTRNWGTLSTSGNGWSLELKSISWNGRPEKYDIRAWSPDKSKMGKGVTLTRAEI
VALRDLLNSMSLDPY

> Tp0031 TPANIC_RS00155 WP_010881480.1 (RANK 17/68)
25 amino acid peptide
MDLGQRVVRVIPLAPLPVRVYNAGGLRVDFFPRFFGRSPQGVGVGFARLKLSASVGSNGFRLTRAVWIFW
LCFLVSGLSRAFLVYFLSVIRI

> Tp0676 TPANIC_RS03350 WP_010882121.1 (RANK 52/68)
16 / 25 amino acid peptide
MRCSHNWDDPPPLFGAVSYGMQEGAGRGVRREARDTPCRGTAEGLATSQPEDGETRAALQRIDHLDTQLL
QLERDLAHYLEMAELPDPFSEN

> TPANIC_RS05520 WP_013945579.1 (RANK 40/68)
18 / 28 amino acid peptide
MIFMRRSGDFAREAVVGAVLVGVLLMGAAVFALLSPMEYGCGLGWAQDALRFLRGAAPVGGIFAGFISLL
VGVANMRDRAPTRKLPERVDKGAEG

> Tp0666 TPANIC_RS03295 WP_010882111.1 (RANK 47/68)
7 amino acid peptide
MQKGSGASSVMTLYEYYLIFPDGECREISGPPCERSLLDMNGHPLRVPLSSNRVIAYRVAGKRTVAGGRG
VVGIWYTLEQLDALELLEYVSGPLGQR

> TPANIC_RS05495 WP_041610078.1 (RANK 34/68)
26 amino acid peptide
MHGVIEMVDVVSGFFSNSMSFLRVGAFALSHAVLSFVVFTMTQFVGGYASLWGILVYVFGNGVIIFLEGL
IVAIQAVRLQYYEFFSKFFTKSGSVFAPFRFGYQED

>Tp0220 TPANIC_RS01095 WP_010881668.1 (RANK 25/68)
22 amino acid peptide
MDNINIAKDVRPGCVLLTVTGAVSSYTYGEFESRVHGALKENHVVLDLSGVTAMSSSGLGVLISAYDEGL
KYQRRLCILNPSESVRRAIELTGFSEMFTVIKSLDELD

> Tp0617 TPANIC_RS03050 WP_158296018.1 (RANK 13/68)
25 amino acid peptide
MYDSYHYITIQAPNEGSVCSFEHGGWYVPKTVLSLLRRRKCQDARAESEELGITGICQNYAVPVQLGVQH
YFGAHWGIDATATVSFGVDTKLAKFRIPYTLRVGPVFRT

> Tp0368 TPANIC_RS01800 WP_010881816.1 (RANK 51/68)
9 / 13 amino acid peptide
MRLRGVAGALLGAVVLVALGLMGVWWVFYPKKGDRGAAVAREPVLLHIDPAQMEAADEPLTLPPIERSRE
RMSAWSEQECLRQLEYPTEKAVQALEHANEKRIQQMLEAVP

> Tp0461 TPANIC_RS02255 WP_010881910.1 (RANK 48/68)
9 / 13 amino acid peptide
MLQAAACGAMRGGVLEYTGKMEGMGVRVERVRCEQNLSKTAFARELGIGVEELDAIERGTMPLSRDLTLL
LCVVFAVNRLWLESGEGEPFSASAVSPQETVQPEEEAREPPIPPPRWRG

> Tp0913 TPANIC_RS04490 WP_010882356.1 (RANK 15/68)
10 amino acid peptide
MPKHNKLLGAFGEAYAARWLATRGYIIITRNWRRATGEIDIIAQQDDTIVFVEVKTLRCTSYADLAIIVG
KRKQKRICETAKHFLASAREYNHMCARFDVIVLRSDPFRRQDVDIVHLPHAFEDLV

> Tp0310 TPANIC_RS01535 WP_010881759.1 (RANK 29/68)
14 / 15 / 16 amino acid peptide
MAAICPSSTAKAGGGGISVDPLNAVIVEGNVVPSASARVPEAAVCAFCIQTQRRVQGEGRVHTEVSYFEV
EAWDALARVCAQQVRPGVGLRVVGRLKQDRWQQEDGVRVQRVKIVAEHVEFQTPFVW

> Tp0618 TPANIC_RS03055 WP_014342809.1 (RANK 3/68)
31 amino acid peptide
MWRKCLGKVVLLGCALPCVAARISVSPKLGAYGDARGGPDLWGLCIKATDAEEVSGDPDDTEMEYLPPRY
APETPLVGLDVAFRAENGFLLQLTVDAALTRLMFRGQCLAGYSFRPGGGKYVSVGSGGF

> Tp0700 TPANIC_RS03475 WP_010882145.1 (RANK 42/68)
23 amino acid peptide
MVMAAAPLQNLCYDGLLQVTSGGNISLPVPSNQVIYAHFEHVDATPAEQGQAGVSVSELQILDALVERLI
VQRRVAAEAADMAVQKRQETLLRAAELFSQKQVDETKRRGESLPYTSVEVQGPELFDLRA

> Tp0563 TPANIC_RS02775 WP_010882010.1 (RANK 10/68)
12 amino acid peptide
MSMTSFYERIGAILRDRLNSDEDPFDQWDNRGGKYRTCAGRMERRPPPKKNPPPGPVRVPVPPELVEDFA
VLSVPAGLPLSYCKQSWKRLLKKYHPDVFTCTHTSEQAADIVRRINSSYKRIETWFETGALPTDNKS

> Tp0638 TPANIC_RS03150 WP_010882083.1 (RANK 16/68)
25 amino acid peptide
MGTLRRFPASVLQIALALFLLASGARDLVHVDAGVFNAAVYFLGGLFRGHVAIGVLTLAVSLCCLTAGFF
LLVDFLRPELSCVSAVLALFVVLWALNMVLVDVVGAFGRGKVLQNVSSALEHLHHTAVDLLVLGALIFVR
QHTR

> Tp1032 TPANIC_RS05100 WP_010882476.1 (RANK 4/68)
23 amino acid peptide
MACGENERASTSPPNRAAAARGGRLTLLDGCCVALVLALTAWSGFFVVYRMQGGARTLDIRCGAQRWTYPL
DQERVIRVRGPLGETEIEIRAGAARVCRSPCANGTCIAHPPVQRVGEWNACLPNGVFLYVHGTDAAEPEA
DAVQ

>Tp0222 TPANIC_RS01105 WP_010881670.1 (RANK 35/68)
8 / 31 amino acid peptide
MWKRGVGALLWGMVLLRSFVAYADSQAATGGLLKEIMGEVASIRLESNMLKQQLRERHAESAALKKALQT
LDVKLEKAAKALEESEHALSESKELIETLRSELEILRQRVNALNMRLRRLEITNNVLIGVAVVCGVAAIG
AGIYAAVK

>Tp0867 TPANIC_RS05365 WP_010882310.1 (RANK 22/68)
19 / 25 amino acid peptide
MRWLCRLIGYRYGYAVCGGYRRQGGRCSAQLHAQYTRVSHCVLRIAWEVNGADYELFRLWI

Supplementary Figure S1. List of *T. pallidum* low molecular weight AMP candidates with Glycine-Glycine / Glycine-Alanine pairs located within the first 31 N-terminal residues. N-terminal peptides (underlined) with Glycine-Glycine (turquoise highlight) and Glycine-Alanine (green highlight) pairs are indicated. The locus tags (Tp [old locus tag] and TPANIC [new locus tag]), accession number (WP_), predicted AMP ranking, and length of the N-terminal Glycine-Glycine- / Glycine-Alanine-containing peptides are listed for each *T. pallidum* AMP candidate.
