# Supplementary material for: Identification and Functional Characterization of Peptides With Antimicrobial Activity From the Syphilis Spirochete, Treponema pallidum
Source: Front Microbiol. 2022 May 3;13:888525. doi: 10.3389/fmicb.2022.888525 (PMC9200625; doi:10.3389/fmicb.2022.888525)
Supplement: Supplementary file 8 [file Data_Sheet_2.PDF]

Supplementary Figure S2

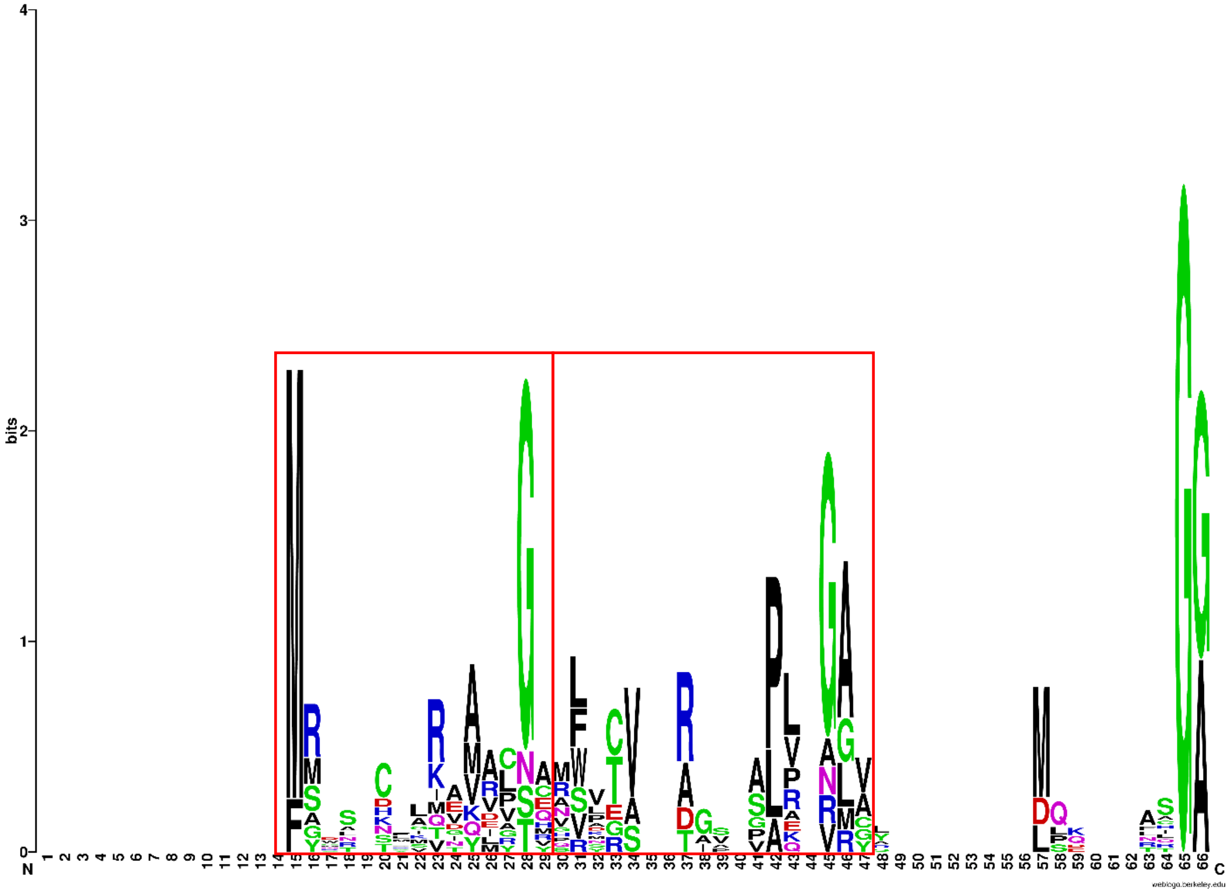

Supplementary Figure S2. Protein homology analysis of 24 *T. pallidum* AMP candidates with N-terminal Glycine-Glycine / Glycine-Alanine pairs. WebLogo graphical representation of the Clustal Omega multiple protein sequence alignment corresponding to the N-terminal regions of 24 *T. pallidum* AMP candidates that contain Glycine-Glycine and/or Glycine-Alanine pairs within the first 31 residues. Two regions with similarity to the double-glycine leader peptide motif from Gram-negatives (M[R/K]ELX$_3$E[I/L]X$_2$[I/V]XG[G/A]) are shown (red rectangles).
