# Supplementary material for: Identification and Functional Characterization of Peptides With Antimicrobial Activity From the Syphilis Spirochete, Treponema pallidum
Source: Front Microbiol. 2022 May 3;13:888525. doi: 10.3389/fmicb.2022.888525 (PMC9200625; doi:10.3389/fmicb.2022.888525)
Supplement: Supplementary file 9 [file Data_Sheet_3.PDF]

Supplementary Figure S3

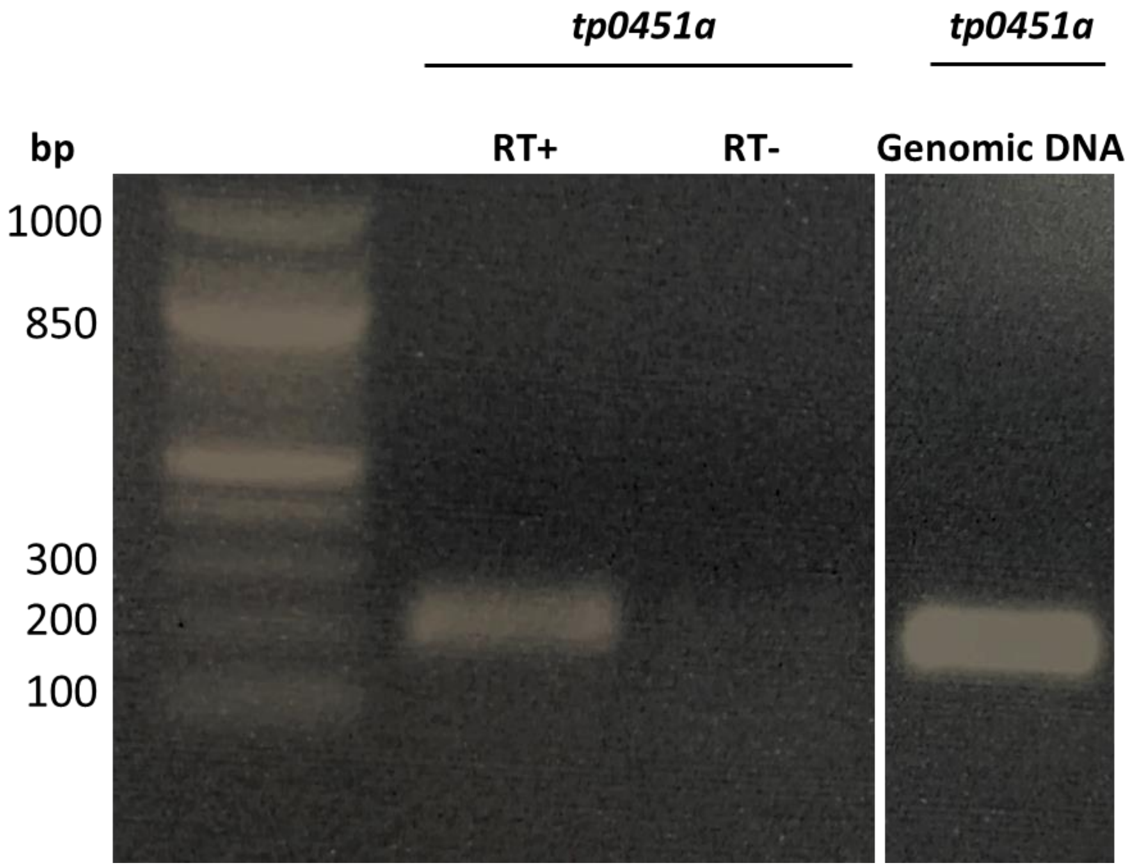

Supplementary Figure S3. RT-PCR analysis of *tp0451a*. Expression of *tp0451a* was confirmed at the transcript level by analysing *T. pallidum* RNA by RT-PCR. The cDNA product amplified from the *tp0451a* primer pair (RT+) and the PCR product of the same size amplified from *T. pallidum* genomic DNA are shown. In comparison, only a faint amplicon was detected when reverse transcriptase was omitted from the RT-PCR reaction (RT−) indicating that the strong 198 base pair product from the RT+ reaction was amplified from RNA and not contaminating DNA.
