# Supplementary material for: Identification and Functional Characterization of Peptides With Antimicrobial Activity From the Syphilis Spirochete, Treponema pallidum
Source: Front Microbiol. 2022 May 3;13:888525. doi: 10.3389/fmicb.2022.888525 (PMC9200625; doi:10.3389/fmicb.2022.888525)
Supplement: Supplementary file 10 [file Data_Sheet_4.PDF]

Supplementary Figure S4

A

Tp0451a
MGCGSHCNCNVGYHRSLHCYGNELHGKQCGFSRCGEGFPAFVAVLMMPFSYSIAEG
VMWGIIAYVILNAVIGRARAITHTWGIWCRWGKVWRRS

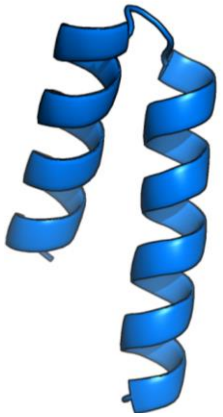

Scoring function	Tp_0451a
Confidence (%)	83.35
QMEAN	0.72
PROSA	-0.69
DOPE	5.77
GA341	0.91
Ramachandran	
% of residues in favoured region	98
% of residues in allowed region	2

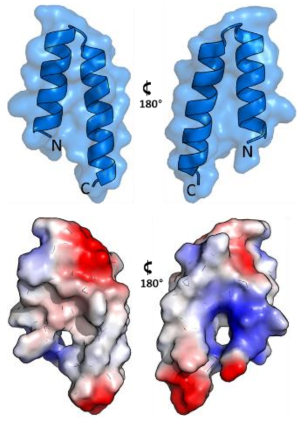

B

Helix I (^{36}EGFPAFVAVLMMPFSY^{51})

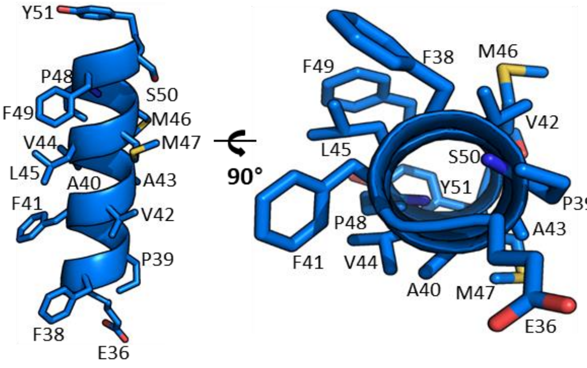

Helix II (^{52}SIAEGVMWGIIAYVILNAVI^{71})

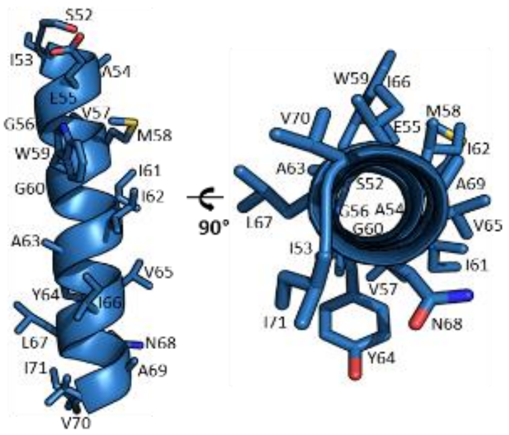

Supplementary Figure S4. Structure modeling of the central region of Tp0451a. (A) Top: Full-length sequence of Tp0451a with modeled residues (E36-I71) highlighted in green. Bottom left: Ribbon structure of Tp0451a E36-I71. Bottom middle: Table showing the scoring functions of the Tp0451a model. Bottom right: Surface image of Tp0451a and charge distribution (red: negatively charged residues; blue: positively charged residues). **(B)** Left: Orthogonal view of Helix 1. Right: Orthogonal view of Helix II. Note the high hydrophobicity of both helices.
