# Supplementary material for: Identification and Functional Characterization of Peptides With Antimicrobial Activity From the Syphilis Spirochete, Treponema pallidum
Source: Front Microbiol. 2022 May 3;13:888525. doi: 10.3389/fmicb.2022.888525 (PMC9200625; doi:10.3389/fmicb.2022.888525)
Supplement: Supplementary file 11 [file Data_Sheet_5.PDF]

Supplementary Figure S5

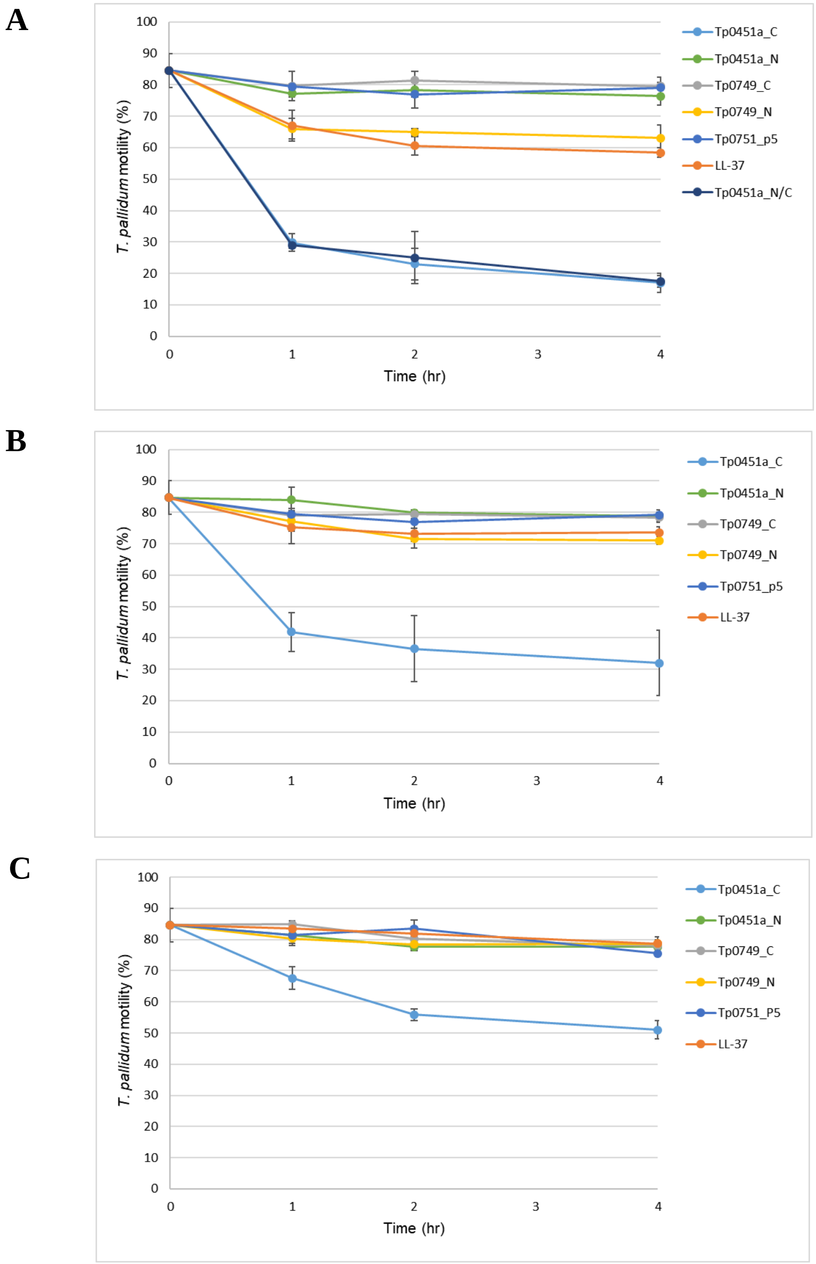

Supplementary Figure S5. AMPCCR susceptibility testing of *T. pallidum*. In vitro-cultured *T. pallidum* was incubated with the four treponemal peptides (Tp0451a_N, Tp0451a_C, Tp0749_N, and Tp0749_C), and the negative (Tp0751_p5) and positive (LL-37) control peptides at **(A)** 64 µg/mL, **(B)** 16 µg/mL, and **(C)** 4 µg/mL. An equimolar mix of Tp0451a_N and Tp0451a_C (Tp0451a_N/C: 21.6µM [85µg/mL Tp0451a_N and 64µg/mL Tp0451a_C]) was also incubated with *T. pallidum* (included in panel **A**). Darkfield microscopy was used to monitor *T. pallidum* viability by counting motile treponemes at 0, 1, 2, and 4 hours post co-incubation. Average *T. pallidum* motility from two experiments are presented with bars indicating standard error.
