# Supplementary material for: Identification and Functional Characterization of Peptides With Antimicrobial Activity From the Syphilis Spirochete, Treponema pallidum
Source: Front Microbiol. 2022 May 3;13:888525. doi: 10.3389/fmicb.2022.888525 (PMC9200625; doi:10.3389/fmicb.2022.888525)
Supplement: Supplementary file 12 [file Data_Sheet_6.PDF]

Supplementary Figure S6

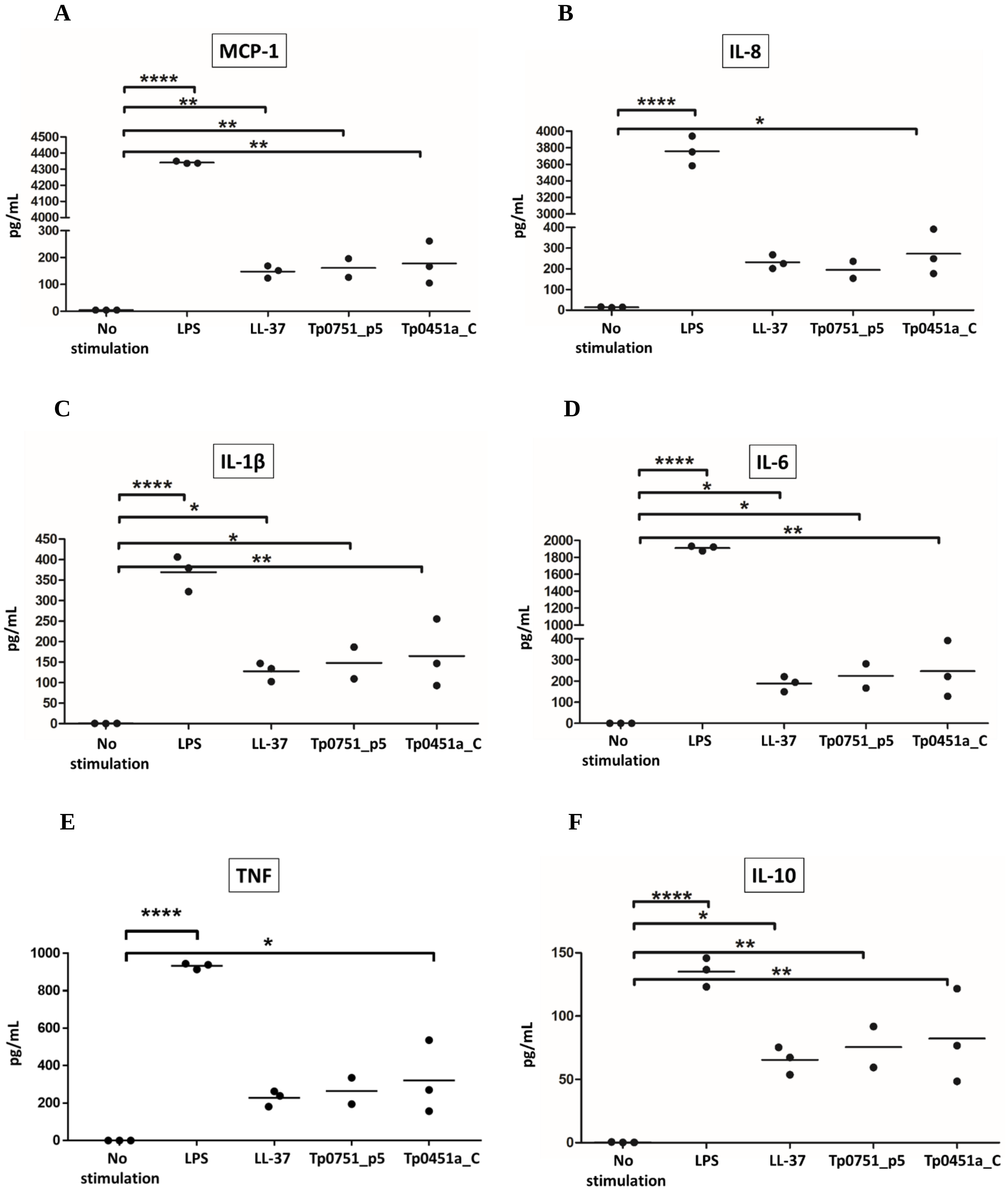

Supplementary Figure S6. Cytokine expression analysis of monocytes following exposure to the *T. pallidum* AMP Tp0451a_C. THP-1 monocytes were stimulated with or without LPS, LL-37, Tp0751_p5, or Tp0451a_C) and analyzed for expression of (A) MCP-1, (B) IL-8, (C) IL-1β, (D) IL-6, (E) TNF, and (F) IL-10. Each data point is representative of cells from one well of a 12-well plate. Data shown is representative of three independent experiments. A Dunnett's multiple comparisons test was used for normally distributed data and a Dunn's multiple comparisons test was used for data that was not normally distributed. For statistical analyses, mean values from each peptide were compared to the mean of the unstimulated control (No stimulation). Significance is represented by *p*-values (* = $p \leq 0.05$, ** = $p \leq 0.01$, *** = $p \leq 0.001$, **** = $p < 0.0001$, no asterisk = not significant).
