# Supplementary material for: Identification and Functional Characterization of Peptides With Antimicrobial Activity From the Syphilis Spirochete, Treponema pallidum
Source: Front Microbiol. 2022 May 3;13:888525. doi: 10.3389/fmicb.2022.888525 (PMC9200625; doi:10.3389/fmicb.2022.888525)
Supplement: Supplementary file 13 [file Data_Sheet_7.PDF]

Supplementary Figure S7

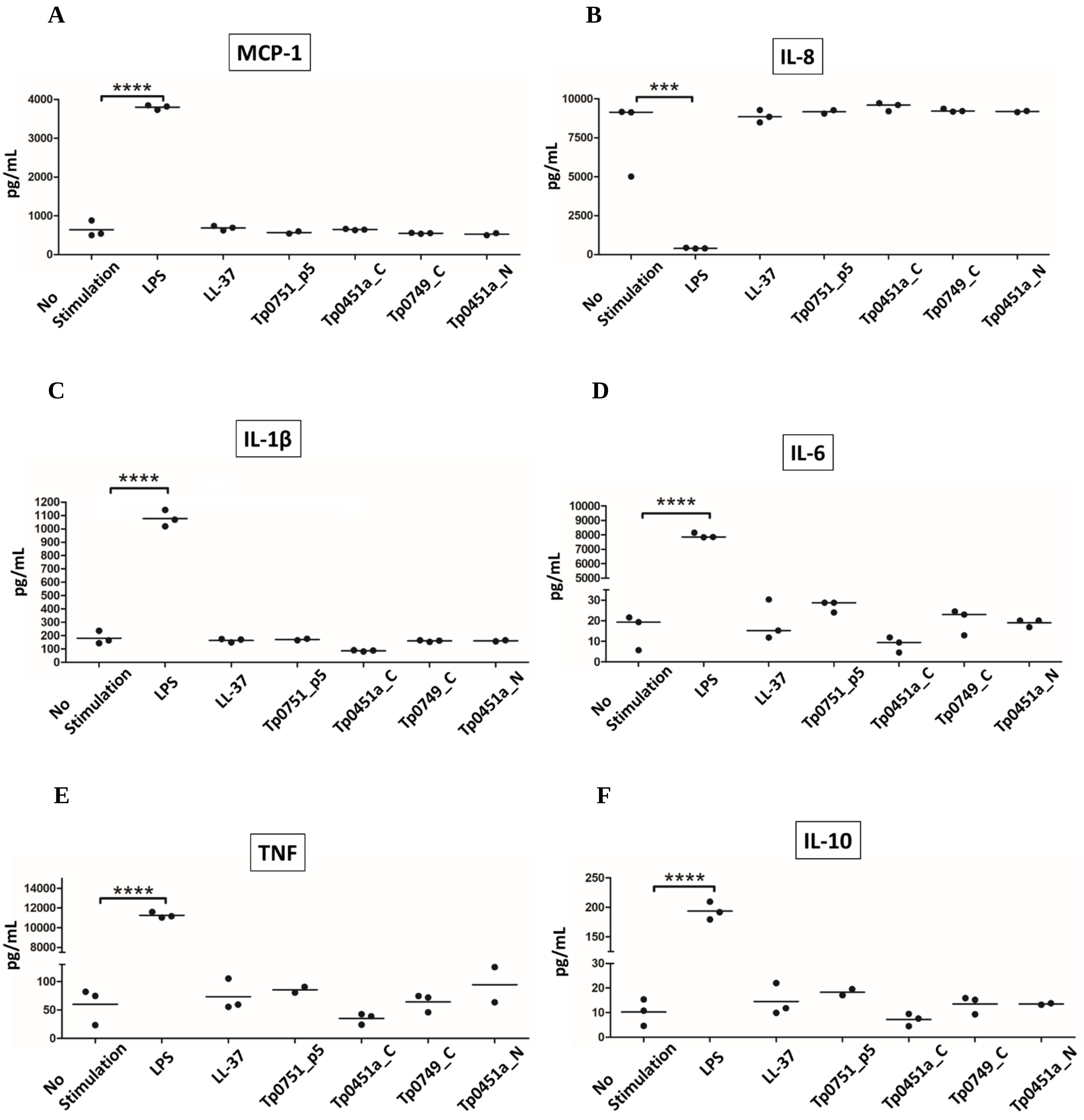

Supplementary Figure S7. Cytokine expression analysis of macrophages following exposure to the *T. pallidum* AMP Tp0451a_C, Tp0749_C, and Tp0451a_N. THP-1 macrophages were stimulated with or without LPS, LL-37, Tp0751_p5, Tp0451a_C, Tp0749_C, or Tp0451a_N and analyzed for expression of (A) MCP-1, (B) IL-8, (C) IL-1β, (D) IL-6, (E) TNF, and (F) IL-10. Each data point is representative of cells from one well of a 12-well plate. Data shown is from one independent experiment. A Dunnett's multiple comparisons test was used for normally distributed data and a Dunn's multiple comparisons test was used for data that was not normally distributed. For statistical analyses, mean values from each peptide were compared to the mean of the unstimulated control (No stimulation). Significance is represented by *p*-values (* = $p \leq 0.05$, ** = $p \leq 0.01$, *** = $p \leq 0.001$, **** = $p < 0.0001$, no asterisk = not significant).
