# Supplementary material for: Identification and Functional Characterization of Peptides With Antimicrobial Activity From the Syphilis Spirochete, Treponema pallidum
Source: Front Microbiol. 2022 May 3;13:888525. doi: 10.3389/fmicb.2022.888525 (PMC9200625; doi:10.3389/fmicb.2022.888525)
Supplement: Supplementary file 14 [file Data_Sheet_8.PDF]

Supplementary Figure S8

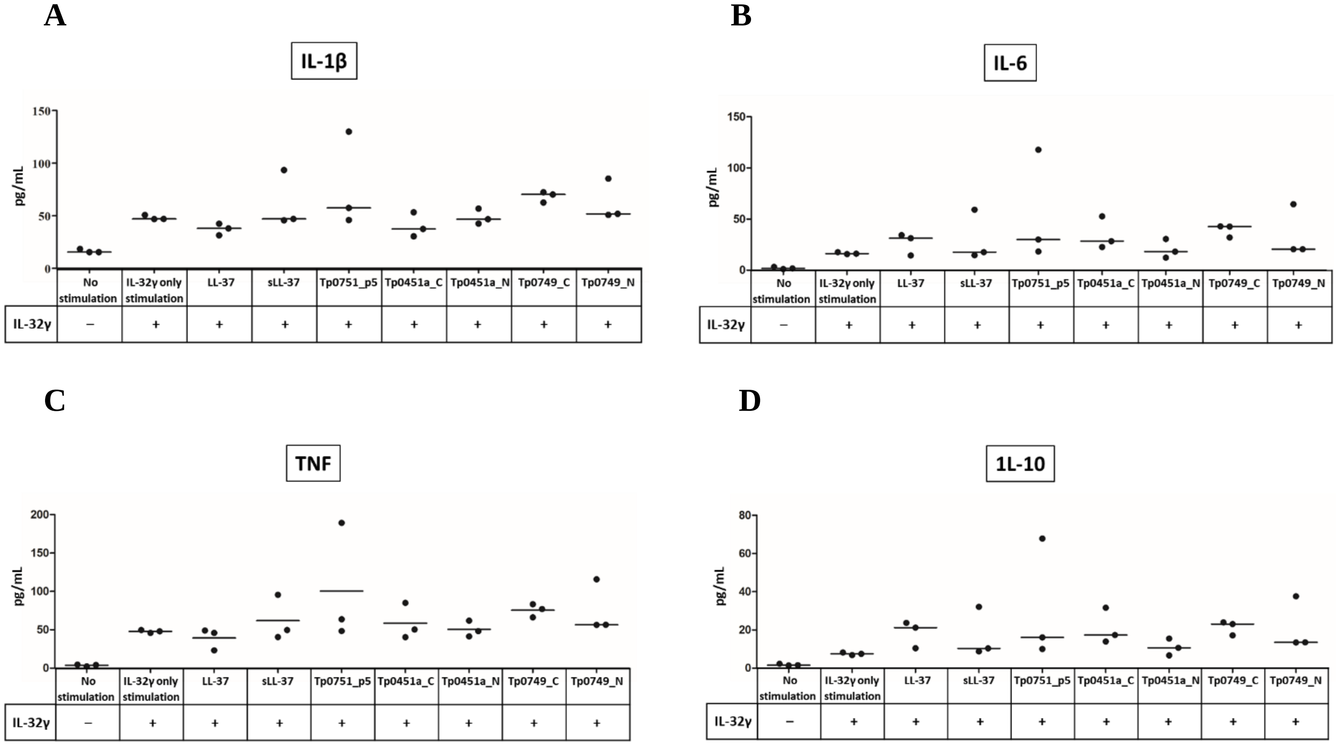

Supplementary Figure S8. Cytokine expression analysis of IL-32γ co-stimulated macrophages. THP-1 macrophages were co-stimulated with or without IL-32γ and LL-37, sLL-37, Tp0751_p5, Tp0451a_C, Tp0451a_N, Tp0749_C, or Tp0749_N and analyzed for expression of **(A)** IL-1β, **(B)** IL-6, **(C)** TNF, and **(D)** IL-10. Each data point is representative of cells from one well of a 12-well plate. Data shown is representative of three independent experiments. A Dunnett's multiple comparisons test was used for normally distributed data and a Dunn's multiple comparisons test was used for data that was not normally distributed. For statistical analyses, mean values from each peptide were compared to the mean of the unstimulated control (No co-stimulation, IL-32γ (+) column). No significant differences in cytokine expression were observed.
